# Physical Activity Types and Sarcopenia Components Among Middle-Aged and Older People: A Cross-Sectional Study

**DOI:** 10.1007/s00223-026-01492-z

**Published:** 2026-02-12

**Authors:** Jiawei Li, Yahong Wu, Artemis Gkitakou, Elizabeth Benz, Carolina Medina-Gomez, M. Carola Zillikens, Ling Oei, Trudy Voortman, Fernando Rivadeneira, Katerina Trajanoska

**Affiliations:** 1 Department of Internal Medicine, Erasmus MC, University Medical Center, Rotterdam, The Netherlands; 2 Department of Epidemiology, Erasmus MC, University Medical Center, Rotterdam, The Netherlands; 3 Meta-Research Innovation Center at Stanford (METRICS), Stanford University, Stanford, USA; 4 Department of Experimental Medicine, Section of Medical Pathology, Endocrinology and Human Nutrition, Sapienza University of Rome, Rome, Italy; 5 Department of Psychiatry, Amsterdam UMC, Vrije Universiteit Amsterdam, Amsterdam Public Health Research Institute, Amsterdam, The Netherlands; 6 Trimbos Institute, Netherlands Institute of Mental Health and Addiction, Utrecht, The Netherlands; 7 Department of Public and Occupational Health, Amsterdam UMC, Amsterdam, The Netherlands; 8 Department of Internal Medicine, Jan Van Goyen Medical Center, Amsterdam, The Netherlands; 9 Department of Internal Medicine, Division of Endocrinology, Leiden University Medical Center, Leiden, The Netherlands

**Keywords:** Sarcopenia, Body composition, Muscle, Public health, Physical activity

## Abstract

Sarcopenia is a strong predictor of morbidity and mortality in older people, highlighting the need for effective prevention strategies. This study examined how different types of physical activity (PA) relate to sarcopenia and its components. We included 4849 participants (55.8% female, mean age 69.1 ± 8.7 years) from the Rotterdam Study. Sarcopenia was defined following the EWGSOP2 criteria using grip strength (GS) and DXA-derived appendicular lean mass (ALMI). Total PA and its types were categorized into low, moderate, and high tertiles. For activities with participation rates below 60%, the low group had no participation, while the remaining participants were split at the median. Probable sarcopenia was present in 559 (11.5%) participants, while 91 (1.9%) had confirmed sarcopenia. Compared with participants in the moderate PA group, those in the high PA group had lower odds of probable sarcopenia (OR 0.70, 95% CI 0.54–0.90) and confirmed sarcopenia (OR 0.38, 95% CI 0.18–0.75). Higher MET hours week^−1^ from sports, cycling, or heavy-domestic work were associated with higher GS and ALMI. No cycling activity tripled the odds of sarcopenia (OR 3.23, 95% CI 1.60–7.27), whereas the high cycling group showed no significant association compared with the moderate group. The low heavy domestic work group was associated with higher odds of probable sarcopenia compared with the moderate group (OR 1.41, 95% CI 1.01–1.96), whereas the high sports activity group had a reduced risk (OR 0.74, 95% CI 0.55–0.99). In this cross-sectional study, older adults who regularly engage in physical activity, particularly cycling, are less likely to have sarcopenia. Additionally, involvement in sports and heavy domestic work is associated with higher values of sarcopenia components.

**Supplementary Information:**

The online version contains supplementary material available at 10.1007/s00223-026-01492-z.

## Introduction

Sarcopenia, characterized by a progressive age-related decline of skeletal muscle mass, strength, and function, affects around 10% of individuals aged 60 and above [1]. Sarcopenia was recognized as a disease in the International Classification of Diseases (ICD-10-CM) in 2016 and has been identified as a public health priority [2]. It poses significant health risks, including disability [3], morbidity [4], and mortality [5], leading to substantial healthcare burdens [6]. Preventing sarcopenia is crucial for improving quality of life and reducing healthcare costs as the population ages [7].

Physical activity (PA) is widely recognized as a key factor of lifelong health [8, 9], offering a wide range of health benefits [9–11]. Nonetheless, nearly one-third of the global population remains inactive [10]. Consequently, it has been demonstrated that older adults in the general population with low or no engagement in regular physical activity have up to two-fold higher risk of developing sarcopenia compared to physically active individuals [11–15]. Nevertheless, most of these studies are either small-scale (up to 600 participants) or define sarcopenia based on a single criterion, primarily appendicular lean mass (ALM) [16–18]. However, the European Working Group on Sarcopenia in Older People (EWGSOP) revised the definition of sarcopenia [19] and placed muscle strength at the forefront of the sarcopenia definition, as it has been shown that strength is a better predictor of adverse outcomes than muscle mass [20, 21]. To date, several studies and meta-analyses have examined the association between PA and muscle strength [22–24], while others have evaluated exercise interventions for sarcopenia [25, 26] or the impact of PA intensity on sarcopenia [27]. By contrast, fewer studies have investigated how specific PA types, such as walking, cycling, and domestic work, relate to sarcopenia and its components, and those that have often involved limited sample sizes [28].

In this large-scale, cross-sectional, population-based study of middle-aged and older people, we aimed to evaluate the association between various types of habitual physical activities, such as cycling, walking, sport engagement, gardening, heavy and light domestic work, in relation to EWGSOP2-defined sarcopenia and its handgrip and lean mass components.

## Methods

### Study Population

Participants of the Rotterdam Study (RS), an ongoing prospective cohort study since 1990 in Rotterdam, the Netherlands. The study's design and sampling procedures have been described previously [29]. For our analysis, we incorporated data from the fifth visit of the first cohort (RS-I-5, from 2009 to 2011; n = 2147), the third visit of the second cohort (RS-II-3, from 2011 to 2012; n = 1893), and the second visit of the extended cohort (RS-III-2, from 2012 to 2014; n = 3122). Additionally, participants with severe obesity (BMI > 35 were excluded due to the potential inaccuracies in dual-energy X-ray absorptiometry (DXA) measurements. Participants lacking assessments of sarcopenia components and physical activity data were also excluded. The Rotterdam Study has been approved by the Medical Ethics Committee of the Erasmus MC (registration number MEC 02.1015) and by the Dutch Ministry of Health, Welfare and Sport (Population Screening Act WBO, license number 1071272-159521-PG). Participants who did not provide written informed consent were excluded from the analysis.

### Physical Activity

Information on total PA and its various types (PA types) was gathered using the LASA Physical Activity Questionnaire (LASAQ) [30], which includes inquiries about the frequency and duration of activities such as walking, cycling (general bicycling), sports (including cycling as a sport), gardening, and housework. In the sports category, participants were asked to list their two most frequently played sports and the time spent on each. All forms of physical activity were assessed using a single questionnaire. LASAQ has previously been validated, demonstrating reasonable test–retest reliability (0.65–0.75), and correlations with the pedometer and the 7-day activity diary were 0.56 and 0.68, respectively [30], indicating good reliability. The intensity of all reported activities was quantified using Metabolic Equivalents of Task (METs). One MET is the energy expended at rest, about 3.5 ml of oxygen per kg of body weight per minute [31]. An activity with a MET value of 4 uses four times the energy required at rest. This standardizes the measurement of energy expenditure, enabling comparisons of activities of different intensities. MET values are multiplied by weekly activity duration to define MET∙hours∙week-1. Total physical activity (Total PA in METs) was calculated by summing all individual physical activities. Physical activity variables included in our study were total physical activity and its subtypes, including cycling, walking, sports, gardening, heavy domestic work, and light domestic work. The Spearman correlation matrix of PA types is shown in Supplementary Fig. 1.

### Appendicular Lean Mass (ALM) and Appendicular Lean Mass Index (ALMI)

Dual X-ray Absorptiometry (DXA; iDXA, GE Health Care, Chicago, United States; enCORE software, GE Health Care) was used to assess total body and regional (e.g., arms, legs, trunk) body composition parameters, including total body mass, lean and fat mass. A team of trained technicians conducted the measurements in accordance with standard imaging and positioning protocols. ALM (kg) was calculated as the sum of the lean tissue in the arms and legs, measured in kilograms, while ALMI was calculated by dividing the ALM (kg) by the square of the height in meters (m^2^). Appendicular lean mass in this study refers exclusively to lean soft tissue, excluding both bone and fat mass [32].

### Grip Strength (GS)

Muscle strength was assessed using a hydraulic dynamometer to measure maximum GS in kilograms. Participants were instructed to sit with their shoulders relaxed, their bodies aligned, and their forearms neutral, elbows bent at 90 degrees. Holding the dynamometer with their non-dominant hand, participants were instructed to exert maximal grip strength under the technician's guidance. Results from three repeated measurements, along with the hand used, were recorded. The maximum value of the three successive measurements was used for data analysis.

### Probable Sarcopenia and Sarcopenia

Sarcopenia was diagnosed following the updated version of the European Working Group on Sarcopenia in Older People-2 (EWGSOP2) criteria [19] (Table 1). The diagnostic process begins by assessing muscle strength through grip strength measurement. Probable sarcopenia is identified when grip strength falls below specific reference values (male < 27 kg; female < 16 kg). If individuals with low grip strength also exhibit low ALMI (male < 7 kg/m^2^; female < 5.5 kg/m^2^), the diagnosis of sarcopenia is confirmed.

**Table 1:** European Working Group on Sarcopenia in Older People 2(EWGSOP2) criteria​ for sarcopenia

	Males	Females
Grip strength, kg	< 27	< 16
Appendicular skeletal muscle mass divided by height^2^, kg/m^2^	< 7	< 5.5
Gait speed, m/sec	< 0.8	< 0.8

*Kg* kilograms; *m* meters; *s* seconds

Probable sarcopenia is identified when grip strength falls below the cutoff points. If individuals with low grip strength also exhibit low ALMI, the diagnosis of sarcopenia is confirmed. If gait speed also falls below the reference value, participants are classified as having severe sarcopenia

### Covariates

We adjusted models for the following covariates: sex, age (years), BMI (kg/m2), alcohol consumption (g/day), educational level, smoking status (never, past, and current), and Rotterdam Study sub-cohort (RS cohort). The covariate data was obtained from the same cohort visits at which PA and the muscle strength and mass parameters were assessed. Alcohol consumption and smoking data was derived from home interviews.

### Statistical Analysis

We analyzed the associations between total PA and PA types (exposures) with the two components of sarcopenia (GS and ALMI) and sarcopenia (outcomes) using regression analysis. The continuous values of PAs, ALMI, and GS are expressed in units of MET∙hours∙week^−1^, kg/m^2^, and kg, respectively. We further categorized the exposures into three intensity groups to assess trends across levels. Total PA and walking were categorized according to their tertile distribution in our study population into three groups “Low” (0 to 33.3%), “Moderate” (33% to 66%) and “High” (> 66%); PA types that were not prevalent in more than 60% of the population, including cycling, sports, gardening, heavy domestic work and light domestic work, were categorized into three groups as follows, no participation (“Low”), below the median participants (“Moderate”) and above the median (“High”) [33–35] (Supplementary Table 1). In addition, ALMI and GS were categorized as “Low” or “High” according to the EWGSP2 cutoff point recommendations, as previously described.

Linear regression models were fitted to assess the associations between total PA and PA types (per 10 MET*hours/week and comparing categories) with continuous ALMI and GS. Logistic regression models were fitted to assess the associations between total PA and PA types with binary ALMI, GS, and sarcopenia status. We used the “moderate activity” group as a reference to better show the direction and effect size across the different categories. The basic model (Model 0) was adjusted for sex (m/f), age (y), and RS cohort. Model 1 was additionally adjusted for BMI (kg/m2), alcohol intake (grams per day), smoking (never, former, current), and education level. Model 2 was additionally adjusted for the other PA types. To prevent overfitting, we checked the models for multicollinearity. As a sensitivity analysis, we performed a sex-stratified analysis. All analyses were conducted using R software (v. 4.3.3).

## Results

### Participant Characteristics

The combined sample size was 4849 participants, and the complete sample size after excluding participants with missing data on relevant covariates was 4765 (Supplementary Fig. 2). The mean (± SD) age of the study participants was 69.1 years (± 8.7), and 55.8% (n = 2707) were female. The mean (± SD) BMI was 26.8 (± 3.4) kg/m^2^. Average alcohol intake per day was 7.6 (± 8.2) grams. Among all participants, 561 (11.6%) were current smokers, 2590 (53.6%) were former smokers, and 1681 (34.8%) were never smokers. Among all participants, 11.5% (n = 559) had low grip strength, indicative of probable sarcopenia, while 6.3% (n = 304) had low ALMI. Overall, 1.9% (n = 91) of participants had both low GS and low ALMI and were confirmed with sarcopenia (Table 2).

**Table 2:** Baseline Rotterdam Study participant characteristics, overall and by groups of total physical activity^a^

	Overall	Groups of total physical activity			*P*-value^e^
		Low	Moderate	High	
Sample size, n	4849	1619	1614	1616	
*Sex, n, (%)*					
Males	2142 (44.2)	886 (54.7)	836 (51.8)	985 (61.0)	< 0.001
Females	2707 (55.8)	733 (45.3)	778 (48.2)	631 (39.0)	
Age, year	69.1 (8.7)	70.8 (9.5)	68.2 (8.8)	68.3 (7.6)	< 0.001
Body mass index (BMI), kg/m^2^	26.8 (3.4)	27.2 (3.5)	26.93 (3.4)	26.3 (3.3)	< 0.001
The Rotterdam Study Sub-cohorts^b^, n, (%)					
RSI-5	1157 (23.9)	518 (32.0)	341 (21.1)	298 (18.4)	< 0.001
RSII-3	1354 (27.9)	425 (26.3)	435 (27.0)	494 (30.6)	
RSIII-1	2338 (48.2)	676 (41.7)	838 (51.9)	824 (51.0)	
Alcohol intake, g/day	7.6 (8.2)	7.55 (7.6)	7.21 (8.5)	8.11 (8.5)	0.007
Smoking status, n, (%)					
Never	1681 (34.8)	528 (32.7)	543 (33.7)	610 (37.9)	0.010
Past	2590 (53.6)	849 (52.7)	880 (54.7)	861 (53.5)	0.585
Current	561 (11.6)	235 (14.6)	187 (11.6)	139 (8.6)	< 0.001
Educational level, n, (%)					
Primary education	358 (7.5)	139 (8.7)	110 (6.9)	109 (6.8)	< 0.001
Lower education	1861 (38.8)	689 (43.0)	531 (33.3)	641 (40.2)	
Intermediate education	1447 (30.2)	457 (28.5)	506 (31.7)	484 (30.4)	
Higher education	1126 (23.5)	319 (19.9)	447 (28.0)	360 (22.6)	
Physical activities, MET*h*week-1					
Total physical activity	42.0 (16.8, 80.9)	11.0 (6.0, 16.8)	42.0 (31.5, 53.0)	99.4 (80.9, 133.3)	< 0.001
Sports	5.3 (0, 19.0)	0 (0, 0)	6.8 (0, 15.5)	23.27 (10.2, 44.1)	< 0.001
Cycling	0.7 (0, 6.0)	0 (0, 2.0)	1.0 (0, 6.0)	4.0 (0, 12.0)	< 0.001
Heavy domestic work	0 (0, 8.6)	0 (0, 0)	2.15 (0, 6.5)	10.8 (4.3, 19.4)	< 0.001
Light domestic work	4.9 (0, 19.6)	0.00 (0, 0)	7.0 (0, 17.5)	28.0 (13.1, 39.7)	< 0.001
Gardening	0 (0, 2.0)	0.00 (0, 0)	0 (0, 2.0)	0 (0, 8.0)	< 0.001
Walking	7.5 (3.5, 15.0)	6.0 (3.0, 10.5)	7.9 (3.4, 15.8)	11.3 (5.0, 21.0)	< 0.001
Appendicular lean mass index (ALMI)^c^, kg/m^2^	7.4 (1.1)	7.3 (1.1)	7.5 (1.2)	7.4 (1.1)	< 0.001
Low, n, %	304 (6.3)	144 (8.9)	85 (5.3)	75 (4.6)	< 0.001
High, h, %	4545 (93.7)	1475 (91.1)	1529 (94.7)	1541 (95.4)	
Maximum grip strength (GS)^c^, kg	28.6 (10.2)	27.3 (10.32)	29.7(10.6)	28.7 (9.4)	< 0.001
Low, n, %	559 (11.5)	267 (16.5)	172 (10.7)	120 (7.4)	< 0.001
High, n, %	4290 (88.5)	1352 (83.5)	1442 (89.3)	1496 (92.6)	
Sarcopenia^c^, n, (%)					
No	4758 (98.1)	1570 (97.0)	1584 (98.1)	1604 (99.3)	< 0.001
Yes	91 (1.9)	49 (3.0)	30 (1.9)	12 (0.7)	

^a^Physical activity types were expressed as median and interquartile range (IQR); The remaining continuous variables were expressed as mean with standard deviation (SD); Categorical variables were expressed with as counts with percentages (n, %)

^b^RS-I-5: the fifth visit of the first cohort; RS-II-3: the third visit of the second cohort; RS-III-1: the first visit of the third cohort

^c^ALMI (Low and High), GS (Low and High), Probable sarcopenia and sarcopenia were categorized according to the EWGSOP2 criteria (detailed in Table 1)

^d^GS Low is also defined as probable sarcopenia

^e^*P*-values were calculated among three groups using the ANOVA method

### Association Between Types of Physical Activity and Sarcopenia and Its Components

#### Association Between Physical Activities and ALMI

Higher total PA was associated with higher ALMI values (β: 0.01, 95% CI 0.01–0.02 per 10 MET∙hours∙week-1 increase). Compared to the moderate total PA group, the high total PA group was associated with higher ALMI values (β: 0.06, 95% CI 0.03 to 0.10), whereas the low total PA group was associated with lower ALMI values (β: − 0.14, 95% CI − 0.18 to − 0.10). When applying the EWGSOP cut-offs for low ALMI, each 10 MET∙hours∙week^−1^ increase in total PA was associated with 7% higher odds of having high ALMI (OR 1.07, 95% CI 1.04 to 1.11). Moreover, participants in the low total PA group had 42% higher odds of having low ALMI (OR 0.58, 95% CI 0.42 to 0.81), while those in the high total PA group did not differ from the middle total PA group (OR 1.36, 95% CI 0.94 to 1.97) (Table 3).

**Table 3:** Associations between total physical activity and specific physical activity types with appendicular lean mass index

	Continuous		Categorical (High vs Low)^c^	
	Appendicular lean mass index (Model 1^a^)	Appendicular lean mass index (Model 2^b^)	Appendicular lean mass index (Model 1^a^)	Appendicular lean mass index (Model 2^b^)
	β [95% CI]	β [95% CI]	Odds ratio^e^ [95% CI]	Odds ratio [95% CI]
Total PA				
Per 10 METh/week^d^	0.01[0.01, 0.02]		1.07[1.04, 1.11]	
	*p* < 0.01		*p* < 0.01	
Groups				
Low	− 0.14[− 0.18, − 0.10]		0.58[0.42, 0.81]	
	*p* < 0.01		*p* < 0.01	
Moderate	ref		ref	
High	0.06[0.03, 0.10]		1.36[0.94, 1.97]	
	*p* < 0.01		*p* = 0.11	
P for trend	*p* < 0.01		*p* < 0.01	
Sports				
Per 10 METh/week^d^	0.02[0.02, 0.03]	0.02[0.01, 0.02]	1.17[1.08, 1.27]	1.13[1.04, 1.23]
	*p* < 0.01	*p* < 0.01	*p* < 0.01	*p* < 0.01
Groups				
Low	− 0.06[− 0.10, − 0.02]	0.04[− 0.03, 0.12]	0.89[0.64, 1.23]	1.75[0.85, 3.51]
	*p* < 0.01	*p* = 0.28	*p* = 0.48	*p* = 0.12
Moderate	ref	ref	ref	ref
High	0.13[0.09, 0.17]	0.09[0.05, 0.13]	1.75[1.19, 2.58]	1.37[0.92, 2.06]
	*p* < 0.01	*p* < 0.01	*p* < 0.01	*p* = 0.13
P for trend	*p* < 0.01	*p* < 0.01	*p* < 0.01	*p* = 0.50
Cycling				
Per 10 METh/week^d^	0.07[0.05, 0.09]	0.06[0.04, 0.07]	1.97[1.53, 2.62]	1.81[1.41, 2.41]
	*p* < 0.01	*p* < 0.01	*p* < 0.01	*p* < 0.01
Groups				
Low	− 0.18[− 0.22, − 0.14]	− 0.15[− 0.19, − 0.11]	0.33[0.22, 0.48]	0.37[0.25, 0.55]
	*p* < 0.01	*p* < 0.01	*p* < 0.01	*p* < 0.01
Moderate	ref	ref	ref	ref
High	0.07[0.03, 0.12]	0.05[0.01, 0.10]	1.20[0.74, 1.95]	1.10[0.67, 1.81]
	*p* < 0.01	*p* = 0.02	*p* = 0.47	*p* = 0.72
P for trend	*p* < 0.01	*p* < 0.01	*p* < 0.01	*p* < 0.01
Heavy domestic work				
Per 10 METh/week^d^	0.03[0.02, 0.04]	0.02[0.01, 0.03]	1.13[1.00, 1.31]	1.04[0.92, 1.20]
	*p* < 0.01	*p* < 0.01	*p* = 0.07	*p* = 0.56
Groups				
Low	− 0.16[− 0.20, − 0.12]	− 0.11[− 0.17, − 0.06]	0.52[0.37, 0.74]	0.47[0.29, 0.77]
	*p* < 0.01	*p* < 0.01	*p* < 0.01	*p* < 0.01
Moderate	ref	ref	ref	ref
High	0.00[− 0.05, 0.04]	− 0.01[− 0.06, 0.04]	1.14[0.73, 1.80]	1.07[0.66, 1.73]
	*p* = 0.91	*p* = 0.70	*p* = 0.56	*p* = 0.79
*P* for trend	*p* < 0.01	*p* < 0.01	*p* < 0.01	*p* = 0.01
Light domestic work				
Per 10 METh/week^d^	0.01[0.00, 0.02]	0.00[− 0.01, 0.00]	1.06[0.98, 1.16]	0.97[0.89, 1.07]
	*p* < 0.01	*p* = 0.36	*p* = 0.14	*p* = 0.57
Groups				
Low	− 0.15[− 0.19, − 0.11]	− 0.04[− 0.12, 0.04]	0.62[0.45, 0.86]	0.90[0.45, 1.84]
	*p* < 0.01	*p* = 0.37	*p* < 0.01	*p* = 0.77
Moderate	ref	ref	ref	ref
High	− 0.04[− 0.08, 0.01]	− − 0.06[− 0.10, − 0.01]	0.92[0.61, 1.41]	0.83[0.53, 1.31]
	*p* = 0.11	*p* = 0.01	*p* = 0.71	*p* = 0.42
P for trend	*p* < 0.01	*p* = 0.02	*p* = 0.02	*p* = 0.19
Gardening				
Per 10 METh/week^d^	0.03[0.01, 0.04]	0.02[0.01, 0.04]	1.21[1.04, 1.44]	1.16[1.00, 1.37]
	*p* < 0.01	*p* < 0.01	*p* = 0.03	*p* = 0.07
Groups				
Low	− 0.08[− 0.12, − 0.04]	− 0.03[− 0.07, 0.01]	0.59[0.39, 0.90]	0.72[0.46, 1.11]
	*p* < 0.01	*p* = 0.17	*p* = 0.02	*p* = 0.14
Moderate	ref	ref	ref	ref
High	0.06[0.00, 0.12]	0.06[0.00, 0.12]	1.33[0.74, 2.42]	1.21[0.66, 2.25]
	*p* = 0.04	*p* = 0.05	*p* = 0.35	*p* = 0.55
P for trend	*p* < 0.01	*p* < 0.01	*p* < 0.01	*p* = 0.03
Walking				
Per 10 METh/week^d^	0.00[− 0.01, 0.01]	0.00[− 0.01, 0.01]	1.00[0.92, 1.10]	0.98[0.90, 1.08]
	*p* = 0.65	*p* = 0.54	*p* = 0.99	*p* = 0.64
Groups				
Low	0.03[− 0.01, 0.07]	0.03[− 0.01, 0.07]	1.12[0.79, 1.58]	1.06[0.74, 1.52]
	*p* = 0.12	*p* = 0.13	*p* = 0.53	*p* = 0.74
Moderate	ref	ref	ref	ref
High	0.03[− 0.01, 0.07]	0.03[− 0.01, 0.07]	0.99[0.71, 1.37]	1.00[0.71, 1.40]
	*p* = 0.19	*p* = 0.11	*p* = 0.94	*p* = 0.98
P for trend	*p* = 0.77	*p* = 0.82	*p* = 0.53	*p* = 0.74

*PA* Physical activity; *CI* Confidence interval

^a^Model 1: adjusted for sex, age, Rotterdam Study Cohort, BMI, alcohol intake, smoking and educational level

^b^Model 2: adjusted for sex, age, Rotterdam Study Cohort, BMI, alcohol intake, smoking, educational level and the other physical activity subtypes

Results of the models only adjusted for sex, age and cohort are present in Supplementary Table 2

^c^Appendicular lean mass index was categorized according to EWGSOP2 criteria​ (Table 1)

^d^The β and odds ratios are per 10 MET*hours*week^−1^ increase in any of the physical activity types for the continuous analysis

^e^The odds ratios represent the odds of being in the high appendicular lean mass index group compared with the low group, across different physical activity types

Sample sizes for the categorical exposures are given in Supplementary Table 1 and for the cross tabs between the categorial exposures and outcomes Supplementary Fig. 3b

Among PA types, cycling, sports, heavy domestic work, and gardening were associated with higher ALMI values, even after adjusting for confounders and other PA types, whereas light domestic work did not show such association (Table 3, Supplementary Table 2). Walking was positively associated with ALMI, but this association was not statistically significant after adjusting for BMI (Supplementary Table 2). Participants in the high sports (β: 0.09, 95% CI 0.05 to 0.13) and high cycling groups (β: 0.05, 95% CI 0.01 to 0.10) had significantly higher ALMI values than those in the moderate group. Furthermore, individuals who did not cycle had significantly lower ALMI values than those in the moderate-cycling group (β: − 0.15, 95% CI − 0.19 to − 0.11). When applying the EWGOP2 cut-offs for low/high ALMI, each 10 MET∙hours∙week-1 increase in cycling and sports was also associated with higher odds of having high ALMI (OR: 1.13, 95% CI 1.04–1.23 for sports and OR: 1.81, 95% CI 1.41–2.41 for cycling). For the cycling and sports groups, a significant difference was observed only between the low and middle cycling groups (OR: 0.37, 95% CI 0.25 to 0.55), whereas no significant differences were observed across the sport groups after adjusting for other PA types.

Furthermore, heavy domestic work was also associated with higher ALMI values (β: 0.02, 95% CI 0.01, 0.03; per 10 MET∙hours∙week-1 units increase), with the low heavy domestic work group significantly associated with lower ALMI values (β: − 0.11, 95% CI − 0.17 to − 0.06) and higher odds of having low ALMI (OR: 0.47, 95% CI 0.29 to 0.77). Gardening was also associated with higher ALMI values (β: 0.02, 95% CI 0.01–0.04). Walking and light domestic work were not associated with ALMI.

#### Association Between Physical Activities and GS and Probable Sarcopenia

Higher total PA was also associated with higher GS values (β: 0.09, 95% CI 0.06–0.13, per 10 MET∙hours∙week-1 increase). Compared with the middle total PA group, the high total PA group was associated with higher GS values (β: 0.49, 95% CI 0.06 to 0.92), whereas the low total PA group was associated with lower GS values (β: − 0.96, 95% CI − 1.39 to − 0.53) (Table 4). When applying EWGSOP2 cut-offs for low GS, referred to as probable sarcopenia, total PA was associated with 5% lower odds of probable sarcopenia (OR: 0.95, 95% CI 0.93 to 0.97, per 10 MET∙hours∙week-1 units increase). When categorized by PA level, the odds of probable sarcopenia were 1.22 (95% CI 0.97, 1.52) in the lowest PA group and 0.70 (95% CI 0.54, 0.90) in the highest PA group, compared with the middle PA group (Table 5).

**Table 4:** Associations between total physical activity and specific physical activity types with maximum grip strength

	Grip strength (Model 1^a^)	Grip strength (Model 2^b^)
	β [CI 95%]	β [CI 95%]
Total PA		
Per 10 METh/week^c^	0.09[0.06, 0.13]	
	*p* < 0.01	
Groups		
Low	− 0.96[− 1.39, − 0.53]	
	*p* < 0.01	
Moderate	ref	
High	0.49[0.06, 0.92]	
	*p* = 0.02	
P for trend	*p* < 0.01	
Sports		
Per 10 METh/week^c^	0.17[0.11, 0.23]	0.15[0.08, 0.21]
	*p* < 0.01	*p* < 0.01
Groups		
Low	− 0.44[− 0.86, − 0.01]	− 0.23[− 1.10, 0.64]
	*p* = 0.05	*p* = 0.60
Moderate	ref	ref
High	0.73[0.27, 1.18]	0.49[0.03, 0.95]
	*p* < 0.01	*p* = 0.04
P for trend	*p* < 0.01	*p* = 0.01
Cycling		
*P*er 10 METh/week^c^	0.35[0.17, 0.53]	0.24[0.06, 0.43]
	*p* < 0.01	*p* = 0.01
Groups		
Low	− 0.64[− 1.06, − 0.21]	− 0.39[− 0.82, 0.04]
	*p* < 0.01	*p* = 0.08
Moderate	ref	ref
High	0.45[− 0.04, 0.94]	0.31[− 0.18, 0.81]
	*p* = 0.07	*p* = 0.21
P for trend	*p* < 0.01	*p* < 0.01
Heavy domestic work		
Per 10 METh/week^c^	0.26[0.14, 0.37]	0.24[0.11, 0.37]
	*p* < 0.01	*p* < 0.01
Groups		
Low	− 1.07[− 1.49, − 0.64]	− 1.33[− 1.95, − 0.70]
	*p* < 0.01	*p* < 0.01
Moderate	ref	ref
High	0.28[− 0.22, 0.78]	0.28[− 0.23, 0.80]
	*p* = 0.28	*p* = 0.28
P for trend	*p* < 0.01	*p* < 0.01
Light domestic work		
Continuous	0.06[− 0.02, 0.15]	− 0.08[− 0.18, 0.01]
	*p* = 0.16	*p* = 0.10
Groups		
Low	− 0.78[− 1.20, − − 0.37]	0.77[− 0.12, 1.67]
	*p* < 0.01	*p* = 0.09
Moderate	ref	ref
High	− 0.19[− 0.68, 0.29]	− 0.49[− 1.00, 0.01]
	*p* = 0.44	*p* = 0.06
P for trend	*p* < 0.01	*p* < 0.01
Gardening		
Per 10 METh/week^c^	0.10[-0.05, 0.25]	0.07[− 0.08, 0.22]
	*p* = 0.20	*p* = 0.35
Groups		
Low	− 0.71[− 1.18, − 0.23]	− 0.49[− 0.97, − 0.01]
	*p* < 0.01	*p* = 0.04
Moderate	ref	ref
High	0.32[− 0.33, 0.96]	0.24[− 0.40, 0.89]
	*p* = 0.34	*p* = 0.46
P for trend	*p* < 0.01	*p* < 0.01
Walking		
Per 10 METh/week^c^	0.08[− 0.03, 0.19]	0.04[− 0.07, 0.14]
	*p* = 0.15	*p* = 0.50
Groups		
Low	0.21[− 0.22, 0.63]	0.20[− 0.22, 0.63]
	*p* = 0.34	*p* = 0.35
Moderate	ref	ref
High	0.23[− 0.20, 0.66]	0.24[− 0.19, 0.66]
	*p* = 0.30	*p* = 0.28
P for trend	*p* = 0.91	*p* = 0.92

*PA* Physical activity; *CI* Confidence interval

^a^Model 1: adjusted for sex, age, Rotterdam Study Cohort, BMI, alcohol intake, smoking and educational level

^b^Model 2: adjusted for sex, age, Rotterdam Study Cohort, BMI, alcohol intake, smoking, educational level and the other subtype physical activities

Results of models only adjusted for sex, age and cohort are present in Supplementary Table 3

^c^The β are per 10 MET*hours*week^−1^ increase in any of the physical activity types for the continuous analysis

Sample sizes for the categorical exposures are given in Supplementary Table 1 and for the cross tabs between the categorial exposures and outcomes Supplementary Fig. 3a

**Table 5:** Associations between total physical activity and specific physical activity types with odds for probable sarcopenia and sarcopenia

	Probable sarcopenia^c^ (Model 1^a^)	Probable sarcopenia (Model 2^b^)	Sarcopenia (Model 1^a^)	Sarcopenia (Model 2^b^)
	Odds ratio [95% CI]	Odds ratio [ 95% CI]	Odds ratio^e^ [ 95% CI]	Odds ratio [ 95% CI]
Total physical activity				
Per 10 METh/week^d^	0.95[0.93, 0.97]		0.88[0.82, 0.94]	
	*p* < 0.01		*p* < 0.01	
Groups				
Low	1.22[0.97, 1.52]		1.34[0.81, 2.25]	
	*p* = 0.09		*p* = 0.26	
Moderate	ref		ref	
High	0.70[0.54, 0.90]		0.38[0.18, 0.75]	
	*p* < 0.01		*p* < 0.01	
*P* for trend	*p* < 0.01		*p* < 0.01	
Sports				
Per 10 METh/week^d^	0.89[0.83, 0.94]	0.91[0.85, 0.96]	0.81[0.67, 0.94]	0.90[0.74, 1.05]
	*p* < 0.01	*p* < 0.01	*p* = 0.02	*p* = 0.26
Groups				
Low	1.09[0.87, 1.36]	1.10[0.67, 1.83]	1.45[0.86, 2.53]	0.67[0.23, 2.17]
	*p* = 0.46	*p* = 0.71	*p* = 0.18	*p* = 0.49
Moderate	ref	ref	ref	ref
High	0.65[0.49, 0.86]	0.74[0.55, 0.99]	0.64[0.30, 1.30]	0.85[0.39, 1.79]
	*p* < 0.01	*p* = 0.04	*p* = 0.23	*p* = 0.68
P for trend	*p* < 0.01	*p* = 0.06	*p* = 0.02	*p* = 0.56
Cycling				
Per 10 METh/week^d^	0.76[0.65, 0.89]	0.82[0.69, 0.95]	0.32[0.15, 0.60]	0.37[0.17, 0.68]
	*p* < 0.01	*p* = 0.01	*p* < 0.01	*p* < 0.01
Groups				
Low	1.23[0.97, 1.59]	1.14[0.89, 1.47]	3.66[1.83, 8.19]	3.23[1.60, 7.27]
	*p* = 0.09	*p* = 0.31	*p* < 0.01	*p* < 0.01
Moderate	ref	ref	ref	ref
High	0.68[0.49, 0.94]	0.73[0.53, 1.01]	0.70[0.23, 1.99]	0.79[0.25, 2.30]
	*p* = 0.02	*p* = 0.06	*p* = 0.51	*p* = 0.67
P for trend	*p* < 0.01	*p* < 0.01	*p* < 0.01	*p* < 0.01
Heavy domestic work				
Per 10 METh/week^d^	0.86[0.77, 0.95]	0.90[0.80, 1.00]	0.50[0.29, 0.76]	0.55[0.30, 0.90]
	*p* < 0.01	*p* = 0.06	*p* < 0.01	*p* = 0.04
Groups				
Low	1.35[1.06, 1.73]	1.41[1.01, 1.96]	1.97[1.10, 3.75]	1.84[0.80, 4.18]
	*p* = 0.02	*p* = 0.04	*p* = 0.03	*p* = 0.15
Moderate	ref	ref	ref	ref
High	0.73[0.52, 1.01]	0.76[0.54, 1.07]	0.67[0.26, 1.60]	0.70[0.26, 1.80]
	*p* = 0.06	*p* = 0.11	*p* = 0.37	*p* = 0.47
P for trend	*p* < 0.01	*p* < 0.01	*p* < 0.01	*p* = 0.06
Light domestic work				
Per 10 METh/week^d^	0.97[0.92, 1.02]	1.03[0.98, 1.09]	0.90[0.77, 1.04]	1.08[0.91, 1.23]
	*p* = 0.21	*p* = 0.21	*p* = 0.18	*p* = 0.34
Groups				
Low	1.25[0.99, 1.58]	0.75[0.45, 1.25]	1.93[1.11, 3.46]	1.37[0.43, 4.06]
	*p* = 0.06	*p* = 0.27	*p* = 0.02	*p* = 0.59
Moderate	ref	ref	ref	ref
High	1.00[0.75, 1.32]	1.15[0.86, 1.54]	1.08[0.50, 2.26]	1.15[0.51, 2.54]
	*p* = 0.99	*p* = 0.35	*p* = 0.84	*p* = 0.73
P for trend	*p* = 0.05	*p* = 0.02	*p* = 0.03	*p* = 0.78
Gardening				
Per 10 METh/weekd	0.94[0.84, 1.03]	0.96[0.87, 1.05]	0.72[0.44, 0.99]	0.78[0.49, 1.04]
	*p* = 0.23	*p* = 0.43	*p* = 0.10	*p* = 0.17
Groups				
Low	1.23[0.91, 1.68]	1.14[0.84, 1.57]	2.50[1.12, 6.67]	2.10[0.92, 5.72]
	*p* = 0.20	*p* = 0.42	*p* = 0.04	*p* = 0.11
Moderate	ref	ref	ref	ref
High	0.76[0.49, 1.17]	0.81[0.52, 1.25]	0.80[0.20, 2.89]	0.88[0.21, 3.26]
	*p* = 0.21	*p* = 0.35	*p* = 0.73	*p* = 0.84
P for trend	*p* = 0.01	*p* = 0.06	*p* = 0.01	*p* = 0.04
Walking				
Per 10 METh/week^d^	0.96[0.89, 1.03]	0.98[0.90, 1.05]	0.92[0.76, 1.08]	0.93[0.76, 1.10]
	*p* = 0.29	*p* = 0.54	*p* = 0.37	*p* = 0.46
Groups				
Low	0.90[0.71, 1.14]	0.90[0.71, 1.14]	0.65[0.36, 1.15]	0.71[0.39, 1.28]
	*p* = 0.39	*p* = 0.37	*p* = 0.14	*p* = 0.26
Moderate	ref	ref	ref	ref
High	0.90[0.71, 1.13]	0.91[0.72, 1.15]	0.78[0.46, 1.32]	0.75[0.44, 1.29]
	*p* = 0.37	*p* = 0.42	*p* = 0.35	*p* = 0.31
P for trend	*p* = 0.97	*p* = 0.92	*p* = 0.58	*p* = 0.88

*PA* Physical activity; *CI* Confidence interval

^a^Model 1: adjusted for sex, age, Rotterdam Study Cohort, BMI, alcohol intake, smoking and educational level

^b^Model 2: adjusted for sex, age, Rotterdam Study Cohort, BMI, alcohol intake, smoking, educational level and the other subtype physical activities

Results of models only adjusted for sex, age and cohort are present in Supplementary Table 4

^c^Probable sarcopenia and sarcopenia were categorized according to EWGSOP2 criteria​ (Table 1). Probable sarcopenia was also defined as low grip strength according to EWGSOP2 cut-off points

^d^The β and odds ratios are per 10 MET*hours*week^−1^ increase in any of the physical activity types for the continuous analysis

^e^The odds ratios represent the risk for probable sarcopenia and sarcopenia compared to non-sarcopenic individuals across different physical activity types

Sample sizes for the categorical exposures are given in Supplementary Table 1 and for the cross tabs between the categorial exposures and outcomes Supplementary Fig. 3a

Cycling, sports, and heavy domestic work were also associated with higher GS values (β:0.24, 95% CI 0.06–0.43 for cycling; β:0.15, 95% CI 0.08–0.21 for sports; β:0.24, 95% CI 0.11–0.37 for heavy domestic work) (Table 4, Supplementary Table 4). Participants in the high sports group had significantly higher GS values than those in the moderate group (β: 0.49, 95% CI 0.03–0.95). Furthermore, individuals who did not engage in heavy domestic work (β: − 1.33, 95% CI − 1.95 to − 0.70) and gardening (β: − 0.49, 95% CI − 0.97 to − 0.01) had significantly lower GS values than those in the moderate group. For probable sarcopenia, significant differences were observed between the high and middle sports group (OR: 0.74, 95% CI 0.55 to 0.99) and the low and middle heavy domestic work group (OR: 1.41, 95% CI 1.01 to 1.96), while no significant differences were observed across the gardening group after adjusting for other PA types (Table 5).

#### Association Between Physical Activities and Sarcopenia

Each 10 MET∙hours∙week^−1^ increase in total PA was associated with 12% lower odds of sarcopenia (OR: 0.88, 95% CI 0.82–0.94) (Table 5, Supplementary Table 6). Among the different PA types, only cycling and heavy domestic work were associated with lower odds of having sarcopenia (OR: 0.37, 95% CI 0.17–0.68 for cycling; OR: 0.55, 95% CI 0.30–0.90 for heavy domestic work) after adjusting for all other PA types (Table 5). We do not have sufficient power to interpret the total PA and PA types (Table 5, Supplementary Table 7) in relation to sarcopenia (Supplementary Fig. 3).

#### Sex-Stratified Association Between Physical Activities and ALMI and GS

Total physical activity was positively associated with ALMI and GS values in both male (β: 0.02, 95% CI 0.02–0.03 for ALMI; β: 0.14, 95% CI 0.08–0.20 for GS; per 10 MET hours week⁻^1^ increase) and female participants (β: 0.01, 95% CI 0.007–0.014 for ALMI; β: 0.09, 95% CI 0.05–0.12 for GS; per 10 MET hours week⁻^1^ increase). In both males and females, engaging in sports, cycling, gardening, and heavy domestic work was associated with higher ALMI after adjusting for all other PA types (Supplementary Tables 8 and 9). Furthermore, sports were associated with higher GS in both males and females, even after adjustment for all other PA types (Supplementary Tables 8 and 9). Heavy domestic work was associated with GS only in men, while cycling showed an association with GS only in females (Supplementary Tables 8 and 9).

## Discussion

This population-based study of middle-aged and older adults found that total PA was associated with lower odds of sarcopenia and higher levels of its components. Among the PA types, sports, cycling, and heavy domestic work were associated with higher ALMI and GS values. Gardening and light housework were also positively associated with these components, although to a lesser extent. Whereas walking was not associated with any of the components.

Lifestyle interventions such as PA play a crucial role in preventing and managing the health burden associated with sarcopenia. In our study, we demonstrated that participation in activities such as sports, cycling, heavy domestic work, and gardening was positively associated with ALMI. The positive effect on ALMI was pronounced among individuals who participated at high levels of sports, likely reflecting the greater intensity and muscle-loading demands of these activities. Individuals who did not participate in any sports also had lower ALMI; however, the effect disappeared after accounting for other PA types. On the other hand, ALMI was lower among individuals who did not engage in cycling or heavy domestic work, suggesting that regular participation in these activities might provide benefits for those who otherwise do not. To date, only one study has evaluated the impact of PA types on ALMI, demonstrating a positive association between cycling and ALMI in both sexes, but did not observe similar associations for sports or heavy domestic work [36]. This study differed in the design of the PA questionnaire, the units used to measure PA, and participants’ age, which may have contributed to discrepancies in the findings compared to ours.

Furthermore, previous research has suggested that regular, moderate-intensity PAs such as walking, climbing stairs, cycling, or gardening may help maintain muscular strength [37, 38]. Our findings, however, indicate that not all PA types have the same relationship with muscle strength. While cycling, sports, and heavy domestic work were positively associated with GS, walking, light domestic work, and gardening were not. Interestingly, individuals who did no gardening or light domestic work at all exhibited low GS.

Among all physical activity domains, cycling and heavy domestic work were associated with both probable and confirmed sarcopenia, whereas participation in sports was associated only with probable sarcopenia. When considering levels of PA, high levels of engagement in sports and cycling were linked to lower odds of probable sarcopenia. In contrast, shifting from no cycling to any level of cycling reduces the odds of confirmed sarcopenia, while moving from no heavy domestic work to any level reduces the odds of probable sarcopenia. These findings may reflect a threshold effect, whereby a benefit is achieved when transitioning from no participation to any level of cycling and heavy domestic work. Evidence from isotemporal substitution models indicates that replacing sedentary behavior with moderate-to-vigorous PA is associated with lower odds of sarcopenia, whereas no association is observed when sedentary time is replaced with light-intensity activity [39]. Consistent with these findings, our study showed that moderate- to high-intensity PA types, such as sports, cycling, and heavy domestic work, were associated with probable and confirmed sarcopenia, whereas light-intensity activities, including light domestic work, gardening, and walking, were not. However, reverse causality should always be considered when interpreting these cross-sectional associations.

Cycling is a common mode of commuting in the Netherlands, and half of the participants in our study cycled weekly (51.5%). Cycling showed the most consistent associations with both probable and confirmed sarcopenia, as well as with key measures such as ALMI and GS. This likely reflects the substantial involvement of two major muscle groups (the gluteals and quadriceps) during the repetitive pedaling and powerful leg-drive movements required in cycling. In this study, cycling was performed daily at low to moderate intensity; therefore, the observed association pattern suggests lean-mass maintenance rather than muscle gains [40]. Thus, cycling may serve as a complement to, rather than a replacement for, resistance training, the currently recognized primary treatment for managing sarcopenia among older adults [41].

Finally, numerous studies have explored the relationship between total PA and sarcopenia [12–18, 42], with odds ratios (95%CI) of sarcopenia ranging from 0.15 (0.10–0.54) [14] to 0.73 (0.69–0.77) [15] in cross-sectional studies and from 0.31 (0.20–0.51) [42] to 0.74 (0.55–0.95) [18] in longitudinal studies. We also found that higher total PA levels are associated with reduced odds of sarcopenia, as defined by low ALMI and GS, in accordance with the latest EWGSOP2 recommendations [19].

## Strengths and Limitations

A key strength of the current study is the use of data from the well-designed Rotterdam Study, which includes a large sample of participants aged 50 years and older. Furthermore, the study benefits from comprehensive data collection protocols. Moreover, we used MET-standardized physical activity measurements that account for the duration and intensity of all activities. Although MET standardization does not capture the distinct features of different PA subtypes, this approach provides a consistent and reliable method for quantifying activity intensity across various exercise types. Additionally, our study employed the updated EWGSOP2 criteria, ensuring that the diagnosis of sarcopenia is based on the most current and stringent standards, thereby improving the accuracy of the findings.

However, our study also has limitations. To start, the findings may not be generalizable to regions or populations with different lifestyles and environmental factors, particularly given the unique cycling culture in the Netherlands. Additionally, the number of confirmed cases of sarcopenia is relatively low. The physical activity data were collected through self-report questionnaires, which may be subject to recall and social desirability biases. The cross-sectional study design captures data at a single point in time and may not reflect changes in physical activity, muscle mass, or strength over time. Furthermore, we were also not able to account for the potential reverse causation between PA and sarcopenia. Age-related deterioration in skeletal muscle significantly contributes to impaired physical function in older adults [43–45]. Sarcopenia-related weakness or fragility further reduces PA, creating a feedback loop that worsens sarcopenia and its consequences. However, this does not alter current guidance; resistance-based exercise remains the first-line approach for preventing and treating sarcopenia [41, 46, 47].

## Conclusions

Although our observational study is cross-sectional and cannot establish causality, it provides valuable insights to improve future recommendations on physical activity, including different subtypes, for older adults aiming to preserve muscle mass and strength. The observed associations also suggest that adding regular static or dynamic cycling into daily routines during midlife may help maintain muscle mass and potentially reduce the risk of sarcopenia in later years. However, these findings need to be confirmed through longitudinal and intervention studies.

## Supplementary Information

Below is the link to the electronic supplementary material.

Supplementary file1 (DOCX 808 kb)
