# Supplementary material for: Physical Activity Types and Sarcopenia Components Among Middle-Aged and Older People: A Cross-Sectional Study
Source: Calcif Tissue Int. 2026 Feb 12;117(1):30. doi: 10.1007/s00223-026-01492-z (PMC12901132; doi:10.1007/s00223-026-01492-z)
Supplement: Supplementary file 1 — Supplementary file1 (DOCX 808 kb) [file 223_2026_1492_MOESM1_ESM.docx]

**Contents**

**Supplementary table 1** Cut-off points for population-specific groups for all physical activity types in the Rotterdam Study.

**Supplementary table 2** Associations between total physical activity and specific physical activity types with appendicular lean mass index from basic models using the middle PA group as reference.

**Supplementary table 3** Associations between total physical activity and specific physical activity types with appendicular lean mass index using the low group as reference.

**Supplementary table 4** Associations between total physical activity and specific physical activity types with maximum grip strength from the basic models using the middle group as reference.

**Supplementary table 5** Associations between total physical activity and specific physical activity types with maximum grip strength using the low group as reference.

**Supplementary table 6** Associations between total physical activity and specific physical activity types with probable sarcopenia and sarcopenia from the basic adjusted models using the moderate group as reference.

**Supplementary table 7** Associations between total physical activity and specific physical activity types with odds for probable sarcopenia and sarcopenia using the low group as reference.

**Supplementary table 8** Sex-stratified analysis between total physical activity and specific physical activity types with continuous ALMI and GS in males.

**Supplementary table 9** Sex-stratified analysis between total physical activity and specific physical activity types with continuous ALMI and GS in females.

**Supplementary table 10** Multicollinearity of variables

**Supplementary Figure 1** Spearman correlation matrix between different physical activity types and sarcopenia components.

**Supplementary Figure 2** Flow chart.

**Supplementary Figure 3a** Number and proportion of individuals with probable sarcopenia and sarcopenia allocated in physical activity groups.

**Supplementary Figure 3b** Number and proportion of individuals with low ALMI allocated in physical activity groups**.**

| **Supplementary table 1. Cut-off points for population-specific groups for all physical activity types in the Rotterdam Study.** | | | |
| --- | --- | --- | --- |
| Physical activities,  MET*hours*week^-1^ | **Physical activity groups^a^** | | |
|  | Low | Moderate | High |
| **Total PA** |  |  |  |
| Sample size | N = 1619 | N = 1614 | N = 1616 |
| Cut-off points | 0.05 - 22.5 | 22.5 - 65.75 | > 65.75 |
| **Sports** |  |  |  |
| Sample size | N = 1960 | N = 1427 | N = 1462 |
| Cut-off points | 0 | >0 - 15.22 | > 15.22 |
| **Cycling** |  |  |  |
| Sample size | N = 2394 | N = 1305 | N = 1150 |
| Cut-off points | 0 | >0 - 6 | > 6 |
| **Heavy domestic work** |  |  |  |
| Sample size | N = 2474 | N = 1303 | N = 1072 |
| Cut-off points | 0 | >0 - 8.6 | > 8.6 |
| **Light domestic work** |  |  |  |
| Sample size | N = 2019 | N = 1659 | N = 1171 |
| Cut-off points | 0 | >0 - 19.6 | > 19.6 |
| **Gardening** |  |  |  |
| Sample size | N = 3367 | N = 875 | N = 607 |
| Cut-off points | 0 | >0 - 8 | > 8 |
| **Walking** |  |  |  |
| Sample size | N = 1635 | N = 1594 | N = 1620 |
| Cut-off points | 0 - 4.5 | 4.5 - 12.5 | > 12.5 |

Note. PA: Physical activity.

^a^Total physical activity and walking were categorized by quantile 33.3% and 66.6%; Cycling, sports, gardening, heavy and light domestic work were categorized by “0” and “median”.

| **Supplementary table 2. Associations between total physical activity and specific physical activity types with appendicular lean mass index from basic models using the middle PA group as reference.** | | |
| --- | --- | --- |
|  | **Continuous** | **Categorical (High vs Low)^b^** |
|  | **Appendicular lean mass index**  **(Model 0^a^)** | **Appendicular lean mass index**  **(Model 0^a^)** |
|  | **β [CI 95%]** | **Odds ratio^d^ [CI 95%]** |
| **Total physical PA** |  |  |
| Per 10 METh/week^c^ | 0.004[0.001, 0.009] | 1.04[1.02, 1.07] |
|  | p = 0.03 | p < 0.01 |
| **Groups** |  |  |
| Low | -0.10[-0.16, -0.05] | 0.65[0.48, 0.86] |
|  | p < 0.01 | p < 0.01 |
| Moderate | ref | ref |
| High | -0.04[-0.09, 0.02] | 1.06[0.77, 1.47] |
|  | p = 0.17 | p = 0.71 |
| P for trend | p = 0.17 | p < 0.01 |
| **Sports** |  |  |
| Per 10 METh/week | 0.01[0.00, 0.02] | 1.13[1.06, 1.22] |
|  | p < 0.01 | p < 0.01 |
| **Groups** |  |  |
| Low | -0.04[-0.10, 0.01] | 0.84[0.64, 1.11] |
|  | p = 0.11 | p = 0.23 |
| Moderate | ref | ref |
| High | 0.06[0.01, 0.12] | 1.49[1.06, 2.10] |
|  | p = 0.03 | p = 0.02 |
| P for trend | p < 0.01 | p < 0.01 |
| **Cycling** |  |  |
| Per 10 METh/week | 0.04[0.02, 0.07] | 1.64[1.31, 2.12] |
|  | p < 0.01 | p < 0.01 |
| **Groups** |  |  |
| Low | -0.12[-0.18, -0.07] | 0.44[0.31, 0.61] |
|  | p < 0.01 | p < 0.01 |
| Moderate | ref | ref |
| High | 0.03[-0.03, 0.10] | 1.07[0.69, 1.65] |
|  | p = 0.30 | p = 0.77 |
| P for trend | p < 0.01 | p < 0.01 |
| **Heavy domestic work** |  |  |
| Per 10 METh/week | 0.02[0.00, 0.03] | 1.08[0.97, 1.22] |
|  | p = 0.02 | p = 0.20 |
| **Groups** |  |  |
| Low | -0.06[-0.05, 0.07] | 0.52[0.37, 0.73] |
|  | p = 0.02 | p < 0.01 |
| Moderate | ref | ref |
| High | -0.01[-0.07, 0.06] | 1.12[0.72, 1.75] |
|  | p = 0.84 | p = 0.62 |
| P for trend | p = 0.04 | p < 0.01 |
| **Light domestic work** |  |  |
| Per 10 METh/week | 0.01[0.01, 0.02] | 1.07[0.99, 1.16] |
|  | p < 0.01 | p = 0.11 |
| **Groups** |  |  |
| Low | -0.16[-0.19, -0.12] | 0.65[0.48, 0.88] |
|  | p < 0.01 | p < 0.01 |
| Moderate | ref | ref |
| High | -0.04[-0.09, 0.00] | 1.01[0.68, 1.51] |
|  | p = 0.05 | p = 0.97 |
| P for trend | p = 0.08 | p = 0.02 |
| **Gardening** |  |  |
| Per 10 METh/week | 0.01[-0.01, 0.03] | 1.15[1.01, 1.34] |
|  | p = 0.18 | p = 0.06 |
| **Groups** |  |  |
| Low | -0.09[-0.14, -0.05] | 0.73[0.50, 1.04] |
|  | p < 0.01 | p = 0.10 |
| Moderate | ref | ref |
| High | 0.05[-0.01, 0.11] | 1.32[0.78, 2.26] |
|  | p = 0.09 | p = 0.31 |
| P for trend | p = 0.21 | p < 0.01 |
| **Walking** |  |  |
| Per 10 METh/week | -0.02[-0.03, -0.00] | 0.94[0.89, 1.00] |
|  | p = 0.01 | p = 0.03 |
| **Groups** |  |  |
| Low | 0.09[0.04, 0.15] | 1.30[0.97, 1.76] |
|  | p < 0.01 | p = 0.08 |
| Moderate | ref | ref |
| High | 0.01[-0.05, 0.06] | 0.93[0.71, 1.23] |
|  | p = 0.84 | p = 0.63 |
| P for trend | p < 0.01 | p = 0.03 |

Note. PA: Physical activity; CI: Confidence interval.

^a^Model 0: adjusted for sex, age, and Rotterdam Study Cohort.

^b^Appendicular lean mass index group high and low were categorized according to EWGSOP2 criteria​ (Table 1).

^c^ Continuous measurements.

^d^The odds ratios represent the odds of having a high or low appendicular lean mass index group across different physical activity types.

| **Supplementary table 3. Associations between total physical activity and specific physical activity types with appendicular lean mass index using the low group as reference.** | | | | | | | | |
| --- | --- | --- | --- | --- | --- | --- | --- | --- |
|  | | | | | | | | |
|  | | **Continuous (Per 10 METh/week)** | | | | **Categorical (High vs Low)^d^** | | |
|  | **Appendicular lean mass index**  **(Model 0^a^)** | | **Appendicular lean mass index**  **(Model 1^b^)** | **Appendicular lean mass index**  **(Model 2^c^)** | **Appendicular lean mass index**  **(Model 0^a^)** | | **Appendicular lean mass index**  **(Model 1^b^)** | **Appendicular lean mass index**  **(Model 2^c^)** |
|  | **β [CI 95%]** | | **β [CI 95%]** | **β [CI 95%]** | **Odds ratio^e^[CI 95%]** | | **Odds ratio [CI 95%]** | **Odds ratio [CI 95%]** |
| **Total PA** |  | |  |  |  | |  |  |
| **Groups** |  | |  |  |  | |  |  |
| Low | ref | | ref |  | ref | | ref |  |
| Moderate | 0.10[0.05, 0.16] | | 0.14[0.10, 0.18] |  | 1.55[1.17, 2.06] | | 1.71[1.23, 2.40] |  |
|  | p < 0.01 | | p < 0.01 |  | p < 0.01 | | p < 0.01 |  |
| High | 0.06[0.01, 0.12] | | 0.20[0.16, 0.24] |  | 1.65[1.23, 2.22] | | 2.33[1.65, 3.31] |  |
|  | p = 0.03 | | p < 0.01 |  | p < 0.01 | | p < 0.01 |  |
| P for trend | p = 0.03 | | p < 0.01 |  | p < 0.01 | | p < 0.01 |  |
| **Sports** |  | |  |  |  | |  |  |
| **Groups** |  | |  |  |  | |  |  |
| Low | ref | | ref | ref | ref | | ref | ref |
| Moderate | 0.04[-0.01, 0.10] | | 0.06[0.02, 0.10] | -0.04[-0.12, 0.03] | 1.18[0.90, 1.57] | | 1.13[0.81, 1.57] | 0.57[0.28, 1.17] |
|  | p = 0.11 | | p < 0.01 | p = 0.28 | p = 0.23 | | p = 0.48 | p = 0.12 |
| High | 0.11[0.05, 0.16] | | 0.19[0.15, 0.23] | 0.05[-0.03, 0.13] | 1.76[1.29, 2.43] | | 1.97[1.37, 2.85] | 0.78[0.38, 1.66] |
|  | p < 0.01 | | p < 0.01 | p = 0.21 | p < 0.01 | | p < 0.01 | p = 0.52 |
| P for trend | p < 0.01 | | p < 0.01 | p < 0.01 | p < 0.01 | | p < 0.01 | p = 0.50 |
| **Cycling** |  | |  |  |  | |  |  |
| **Groups** |  | |  |  |  | |  |  |
| Low | ref | | ref | ref | ref | | ref | ref |
| Moderate | 0.12[0.07, 0.18] | | 0.18[0.14, 0.22] | 0.15[0.11, 0.19] | 2.26[1.64, 3.18] | | 3.03[2.08, 4.50] | 2.67[1.82, 4.00] |
|  | p < 0.01 | | p < 0.01 | p < 0.01 | p < 0.01 | | p < 0.01 | p < 0.01 |
| High | 0.16[0.10, 0.22] | | 0.25[0.21, 0.29] | 0.21[0.16, 0.25] | 2.41[1.71, 3.47] | | 3.63[2.44, 5.52] | 2.93[1.94, 4.53] |
|  | p < 0.01 | | p < 0.01 | p < 0.01 | p < 0.01 | | p < 0.01 | p < 0.01 |
| P for trend | p < 0.01 | | p < 0.01 | p < 0.01 | p < 0.01 | | p < 0.01 | p < 0.01 |
| **Heavy domestic work** |  | |  |  |  | |  |  |
| **Groups** |  | |  |  |  | |  |  |
| Low | ref | | ref | ref | ref | | ref | ref |
| Moderate | 0.06[0.01, 0.12] | | 0.16[0.12, 0.20] | 0.11[0.06, 0.17] | 1.53[1.14 2.09] | | 1.91[1.35, 2.72] | 2.12[1.29, 3.46] |
|  | p = 0.02 | | p < 0.01 | p < 0.01 | p < 0.01 | | p < 0.01 | p < 0.01 |
| High | 0.06[-0.002, 0.11] | | 0.16[0.12, 0.20] | 0.11[0.05, 0.17] | 1.54[1.11, 2.18] | | 2.18[1.49, 3.25] | 2.26[1.31, 3.91] |
|  | p = 0.06 | | p < 0.01 | p < 0.01 | p = 0.01 | | p < 0.01 | p < 0.01 |
| P for trend | p = 0.04 | | p < 0.01 | p < 0.01 | p < 0.01 | | p < 0.01 | p = 0.01 |
| **Light domestic work** |  | |  |  |  | |  |  |
| **Groups** |  | |  |  |  | |  |  |
| Low | ref | | ref | ref | ref | | ref | ref |
| Moderate | 0.09[0.04, 0.14] | | 0.15[0.11, 0.19] | 0.04[-0.04, 0.12] | 1.42[1.08, 1.88] | | 1.61[1.16, 2.24] | 1.11[0.54, 2.22] |
|  | p < 0.01 | | p < 0.01 | p = 0.37 | p = 0.01 | | p < 0.01 | p = 0.77 |
| High | 0.04[-0.02, 0.10] | | 0.11[0.07, 0.15] | -0.02[-0.11, 0.07] | 1.41[1.03, 1.96] | | 1.48[1.02, 2.19] | 0.92[0.42, 1.99] |
|  | p = 0.22 | | p < 0.01 | p = 0.64 | p = 0.04 | | p = 0.04 | p = 0.84 |
| P for trend | p = 0.08 | | p < 0.01 | p = 0.02 | p = 0.02 | | p = 0.02 | p = 0.19 |
| **Gardening** |  | |  |  |  | |  |  |
| **Groups** |  | |  |  |  | |  |  |
| Low | ref | | ref | ref | ref | | ref | ref |
| Moderate | -0.01[-0.07, 0.05] | | 0.08[0.04, 0.12] | 0.03[-0.01, 0.07] | 1.36[0.96, 2.00] | | 1.68[1.12, 2.60] | 1.39[0.90, 2.18] |
|  | p = 0.78 | | p < 0.01 | p = 0.17 | p = 0.10 | | p = 0.02 | p = 0.14 |
| High | 0.06[-0.01, 0.13] | | 0.14[0.09, 0.19] | 0.09[0.04, 0.14] | 1.79[1.18, 2.85] | | 2.23[1.40, 3.71] | 1.67[1.02, 2.84] |
|  | p = 0.10 | | p < 0.01 | p < 0.01 | p < 0.01 | | p < 0.01 | p = 0.05 |
| P for trend | p = 0.21 | | p < 0.01 | p < 0.01 | p < 0.01 | | p < 0.01 | p = 0.03 |
| **Walking** |  | |  |  |  | |  |  |
| **Groups** |  | |  |  |  | |  |  |
| Low | ref | | ref | ref | ref | | ref | ref |
| Moderate | -0.09[-0.15, -0.04] | | -0.03[-0.07, 0.01] | -0.03[-0.07, 0.01] | 0.77[0.57, 1.03] | | 0.89[0.63, 1.26] | 0.94[0.66, 1.35] |
|  | p < 0.01 | | p = 0.12 | p = 0.13 | p = 0.08 | | p = 0.53 | p = 0.74 |
| High | -0.09[-0.14, -0.03] | | 0.00[-0.04, 0.03] | 0.00[-0.04, 0.04] | 0.72[0.53, 0.96] | | 0.88[0.62, 1.25] | 0.94[0.65, 1.35] |
|  | p < 0.01 | | p = 0.81 | p = 0.91 | p = 0.03 | | p = 0.48 | p = 0.73 |
| P for trend | p < 0.01 | | p = 0.77 | p = 0.82 | p = 0.03 | | p = 0.53 | p = 0.74 |

Note. PA: Physical activity; CI: Confidence interval.

^a^Model 0: adjusted for sex, age, and Rotterdam Study Cohort.

^b^Model 1: adjusted for sex, age, Rotterdam Study Cohort, BMI, alcohol intake, smoking and educational level.

^c^Model 2: adjusted for sex, age, Rotterdam Study Cohort, BMI, alcohol intake, smoking, educational level and the other subtype physical activities.

^d^Appendicular lean mass index group high and low were categorized according to EWGSOP2 criteria​ (Table 1).

^e^The odds ratios represent the odds of having a high or low appendicular lean mass index group across different physical activity types.

| **Supplementary table 4. Associations between total physical activity and specific physical activity types with maximum grip strength from the basic models using the middle group as reference.** | |
| --- | --- |
|  | **Grip strength (Model 0^a^)** |
|  | **β [CI 95%]** |
| **Total PA** |  |
| Per 10 METh/week^b^ | 0.09[0.06, 0.12] |
|  | p < 0.01 |
| **Groups** |  |
| Low | -0.92[-1.35, -0.50] |
|  | p < 0.01 |
| Moderate | ref |
| High | 0.39[-0.03, 0.82] |
|  | p = 0.07 |
| P for trend | p < 0.01 |
| **Sports** |  |
| Per 10 METh/week | 0.16[0.10, 0.22] |
|  | p < 0.01 |
| **Groups** |  |
| Low | -0.41[-0.83, 0.01] |
|  | p = 0.05 |
| Moderate | ref |
| High | 0.67[0.22, 1.12] |
|  | p < 0.01 |
| P for trend | p < 0.01 |
| **Cycling** |  |
| Per 10 METh/week | 0.32[0.14, 0.50] |
|  | p < 0.01 |
| **Groups** |  |
| Low | -0.56[-0.98, -0.14] |
|  | p < 0.01 |
| Moderate | ref |
| High | 0.43[-0.06, 0.91] |
|  | p = 0.09 |
| P for trend | p < 0.01 |
| **Heavy domestic work** |  |
| Continuous | 0.24[0.12, 0.35] |
|  | p < 0.01 |
| **Groups** |  |
| Low | -1.00[-1.41, -0.58] |
|  | p < 0.01 |
| Moderate | ref |
| High | 0.25[-0.25, 0.74] |
|  | p = 0.33 |
| P for trend | p < 0.01 |
| **Light domestic work** |  |
| Per 10 METh/week | 0.04[-0.04, 0.13] |
|  | p = 0.32 |
| **Groups** |  |
| Low | -0.75[-1.16, -0.34] |
|  | p < 0.01 |
| Moderate | ref |
| High | -0.26[-0.74, 0.22] |
|  | p = 0.29 |
| P for trend | p = 0.01 |
| **Gardening** |  |
| Per 10 METh/week | 0.10[-0.05, 0.25] |
|  | p = 0.17 |
| **Groups** |  |
| Low | -0.62[-1.09, -0.15] |
|  | p < 0.01 |
| Moderate | ref |
| High | 0.37[-0.27, 1.01] |
|  | p = 0.26 |
| P for trend | P = 0.21 |
| **Walking** |  |
| Continuous | 0.08[-0.03, 0.18] |
|  | p = 0.15 |
| **Groups** |  |
| Low | 0.29[-0.13, 0.72] |
|  | p = 0.18 |
| Moderate | ref |
| High | 0.25[-0.17, 0.68] |
|  | p = 0.25 |
| P for trend | p = 0.84 |

Note. PA: Physical activity; CI: Confidence interval.

^a^Model 0: adjusted for sex, age, and Rotterdam Study Cohort.

^b^Continuous measurement.

| **Supplementary table 5. Associations between total physical activity and specific physical activity types with maximum grip strength using the low group as reference.** | | | |
| --- | --- | --- | --- |
|  | **Grip strength (Model 0^a^)** | **Grip strength (model1^b^)** | **Grip strength (model2^c^)** |
|  | **β [CI 95%]** | **β [CI 95%]** | **β [CI 95%]** |
| **Total PA** |  |  |  |
| **Groups** |  |  |  |
| Low | ref | ref |  |
| Moderate | 0.92[0.50, 1.35] | 0.96[0.53, 1.40] |  |
|  | p < 0.01 | p < 0.01 |  |
| High | 1.32[0.89, 1.74] | 1.46[1.02, 1.89] |  |
|  | p < 0.01 | p < 0.01 |  |
| P for trend | p < 0.01 | 0.94[0.51, 1.37] |  |
| **Sports** |  |  |  |
| **Groups** |  |  |  |
| Low | ref | ref | ref |
| Moderate | 0.41[-0.01, 0.83] | 0.44[0.01, 0.86] | 0.23[-0.64, 1.10] |
|  | p = 0.05 | p = 0.05 | p = 0.60 |
| High | 1.08[0.66, 1.50] | 1.16[0.73, 1.59] | 0.72[-0.15, 1.60] |
|  | p < 0.01 | p < 0.01 | p = 0.11 |
| P for trend | p < 0.01 | p < 0.01 | p = 0.01 |
| **Cycling** |  |  |  |
| **Groups** |  |  |  |
| Low | ref | ref | ref |
| Moderate | 0.56[0.14, 0.98] | 0.64[0.21, 1.06] | 0.39[-0.04, 0.82] |
|  | p < 0.01 | p < 0.01 | p = 0.08 |
| High | 0.99[0.55, 1.43] | 1.09[0.64, 1.53] | 0.70[0.24, 1.16] |
|  | p < 0.01 | p < 0.01 | p < 0.01 |
| P for trend | p < 0.01 | p < 0.01 | p < 0.01 |
| **Heavy domestic work** |  |  |  |
| **Groups** |  |  |  |
| Low | ref | ref | ref |
| Moderate | 1.00[0.58, 1.41] | 1.07[0.64, 1.49] | 1.33[0.70, 1.95] |
|  | p < 0.01 | p < 0.01 | p < 0.01 |
| High | 1.24[0.80, 1.69] | 1.34[0.89, 1.79] | 1.61[0.95, 2.27] |
|  | p < 0.01 | p < 0.01 | p < 0.01 |
| P for trend | p < 0.01 | p < 0.01 | p < 0.01 |
| **Light domestic work** |  |  |  |
| **Groups** |  |  |  |
| Low | ref | ref | ref |
| Moderate | 0.75[0.34, 1.16] | 0.78[0.37, 1.20] | -0.77[-1.67, 0.12] |
|  | p < 0.01 | p < 0.01 | p = 0.09 |
| High | 0.49[0.04, 0.94] | 0.59[0.13, 1.05] | -1.27[-2.23, -0.31] |
|  | p = 0.03 | p = 0.01 | p < 0.01 |
| P for trend | p = 0.01 | p < 0.01 | p < 0.01 |
| **Gardening** |  |  |  |
| **Groups** |  |  |  |
| Low | ref | ref | ref |
| Moderate | 0.62[0.15, 1.09] | 0.71[0.23, 1.18] | 0.49[0.01, 0.97] |
|  | p < 0.01 | p < 0.01 | p = 0.04 |
| High | 0.99[0.45, 1.52] | 1.02[0.48, 1.56] | 0.74[0.19, 1.28] |
|  | p < 0.01 | p < 0.01 | p < 0.01 |
| P for trend | P = 0.21 | p < 0.01 | p < 0.01 |
| **Walking** |  |  |  |
| **Groups** |  |  |  |
| Low | ref | ref | ref |
| Moderate | -0.29[-0.72, 0.13] | -0.21[-0.63, 0.22] | -0.20[-0.63, 0.22] |
|  | p = 0.18 | p = 0.34 | p = 0.35 |
| High | -0.04[-0.47, 0.38] | 0.02[-0.41, 0.45] | 0.03[-0.40, 0.47] |
|  | p = 0.85 | p = 0.92 | p = 0.88 |
| P for trend | p = 0.84 | p = 0.91 | p = 0.92 |

Note. PA: Physical activity; CI: Confidence interval.

^a^Model 0: adjusted for sex, age, and Rotterdam Study Cohort.

^b^Model 1: adjusted for sex, age, Rotterdam Study Cohort, BMI, alcohol intake, smoking and educational level.

^c^Model 2: adjusted for sex, age, Rotterdam Study Cohort, BMI, alcohol intake, smoking, educational level and the other subtype physical activities.

| **Supplementary table 6. Associations between total physical activity and specific physical activity types with probable sarcopenia and sarcopenia from the basic adjusted models using the moderate group as reference.** | | |
| --- | --- | --- |
|  | **Probable sarcopenia^b^**  **(Model 0^a^)** | **Sarcopenia**  **(Model 0^a^)** |
|  | **Odds ratio^d^ [CI 95%]** | **Odds ratio [CI 95%]** |
| **Total PA** |  |  |
| Per 10 METh/week ^c^ | 0.95[0.93, 0.97] | 0.90[0.84, 0.96] |
|  | p < 0.01 | p < 0.01 |
| **Groups** |  |  |
| Low | 1.22[0.98, 1.53] | 1.25[0.78 2.03] |
|  | p = 0.08 | p = 0.35 |
| Moderate | ref | ref |
| High | 0.72[0.55, 0.92] | 0.44[0.22, 0.85] |
|  | p < 0.01 | p = 0.02 |
| P for trend | p < 0.01 | p < 0.01 |
| **Sports** |  |  |
| Per 10 METh/week | 0.89[0.84, 0.94] | 0.82[0.68, 0.95] |
|  | p < 0.01 | p = 0.02 |
| **Groups** |  |  |
| Low | 1.11[0.89, 1.38] | 1.41[0.87, 2.35] |
|  | p = 0.37 | p = 0.18 |
| Moderate | ref | ref |
| High | 0.68[0.51, 0.89] | 0.65[0.32, 1.28] |
|  | p < 0.01 | p = 0.22 |
| P for trend | p < 0.01 | p = 0.01 |
| **Cycling** |  |  |
| Per 10 METh/week | 0.77[0.66, 0.89] | 0.36[0.17, 0.66] |
|  | p < 0.01 | p < 0.01 |
| **Groups** |  |  |
| Low | 1.20[0.95, 1.54] | 3.17[1.64, 6.91] |
|  | p = 0.14 | p < 0.01 |
| Moderate | ref | ref |
| High | 0.69[0.50, 0.94] | 0.73[0.24, 2.04] |
|  | p = 0.02 | p = 0.55 |
| P for trend | p < 0.01 | p < 0.01 |
| **Heavy domestic work** |  |  |
| Per 10 METh/week | 0.86[0.77, 0.95] | 0.55[0.33, 0.82] |
|  | p < 0.01 | p < 0.01 |
| **Groups** |  |  |
| Low | 1.35[1.06, 1.72] | 1.76[1.01, 3.25] |
|  | p = 0.02 | p = 0.06 |
| Moderate | ref | ref |
| High | 0.74[0.53, 1.03] | 0.71[0.28, 1.66] |
|  | p = 0.08 | p = 0.44 |
| P for trend | p < 0.01 | p < 0.01 |
| **Light domestic work** |  |  |
| Per 10 METh/week | 0.97[0.92, 1.02] | 0.91[0.79, 1.03] |
|  | p = 0.20 | p = 0.18 |
| **Groups** |  |  |
| Low | 1.24[0.99, 1.57] | 1.72[1.03, 2.97] |
|  | p = 0.06 | p = 0.04 |
| Moderate | ref | ref |
| High | 1.00[0.76, 1.32] | 0.99[0.48, 1.98] |
|  | p = 0.98 | p = 0.97 |
| P for trend | p = 0.05 | p = 0.03 |
| **Gardening** |  |  |
| Per 10 METh/week | 0.93[0.84, 1.02] | 0.74[0.46, 1.01] |
|  | p = 0.18 | p = 0.13 |
| **Groups** |  |  |
| Low | 1.23[0.91, 1.68] | 2.08[0.96, 5.46] |
|  | p = 0.19 | p = 0.09 |
| Moderate | ref | ref |
| High | 0.74[0.48, 1.14] | 0.73[0.18, 2.58] |
|  | p = 0.17 | p = 0.63 |
| P for trend | p < 0.01 | p = 0.02 |
| **Walking** |  |  |
| Per 10 METh/week | 0.97[0.89, 1.04] | 0.99[0.83, 1.13] |
|  | p = 0.36 | p = 0.94 |
| **Groups** |  |  |
| Low | 0.89[0.70, 1.12] | 0.58[0.33, 0.99] |
|  | p = 0.31 | p = 0.048 |
| Moderate | ref | ref |
| High | 0.90[0.72, 1.13] | 0.81[0.50, 1.32] |
|  | p = 0.35 | p = 0.41 |
| P for trend | p = 0.95 | p = 0.27 |

Note. PA: Physical activity; CI: Confidence interval.

^a^Model 0: adjusted for sex, age, and Rotterdam Study Cohort.

^b^Probable sarcopenia and sarcopenia were categorized according to EWGSOP2 criteria​ (Table 1). Probable sarcopenia was also defined as low grip strength according to EWGSOP2 cut-off points.

^c^Continuous measurements.

^d^The odds ratios represent the risk for probable sarcopenia and sarcopenia compared to non-sarcopenic individuals across different physical activity types.

| **Supplementary table 7. Associations between total physical activity and specific physical activity types with odds for probable sarcopenia and sarcopenia using the low group as reference.** | | | | | | |
| --- | --- | --- | --- | --- | --- | --- |
|  | **Probable sarcopenia^d^**  **(Model 0^a^)** | **Probable sarcopenia**  **(Model 1^b^)** | **Probable sarcopenia**  **(Model 2^c^)** | **Sarcopenia**  **(Model 0^a^)** | **Sarcopenia**  **(Model 1^b^)** | **Sarcopenia**  **(Model 2^c^)** |
|  | **Odds ratio^e^ [CI 95%]** | **Odds ratio [CI 95%]** | **Odds ratio [CI 95%]** | **Odds ratio [CI 95%]** | **Odds ratio [CI 95%]** | **Odds ratio [CI 95%]** |
| **Total PA** |  |  |  |  |  |  |
| **Groups** |  |  |  |  |  |  |
| Low | ref | ref |  | ref | ref |  |
| Moderate | 0.82[0.65, 1.02] | 0.82[0.66, 1.03] |  | 0.80[0.49, 1.28] | 0.75[0.44, 1.24] |  |
|  | p = 0.08 | p = 0.09 |  | p = 0.35 | p = 0.26 |  |
| High | 0.58[0.46, 0.74] | 0.57[0.44, 0.73] |  | 0.35[0.18, 0.66] | 0.28[0.14, 0.54] |  |
|  | p < 0.01 | p < 0.01 |  | p < 0.01 | p < 0.01 |  |
| P for trend | p < 0.01 | p < 0.01 |  | p < 0.01 | p < 0.01 |  |
| **Sports** |  |  |  |  |  |  |
| **Groups** |  |  |  |  |  |  |
| Low | ref | ref | ref | ref | ref | ref |
| Moderate | 0.90[0.73, 1.12] | 0.92[0.74, 1.15] | 0.91[0.55, 1.50] | 0.71[0.43, 1.15] | 0.69[0.40, 1.17] | 1.50[0.46, 4.44] |
|  | p = 0.37 | p = 0.46 | p = 0.71 | p = 0.18 | p = 0.18 | p = 0.49 |
| High | 0.61[0.47, 0.79] | 0.60[0.46, 0.78] | 0.67[0.39, 1.14] | 0.46[0.24, 0.84] | 0.44[0.22, 0.83] | 1.28[0.36, 4.11] |
|  | p < 0.01 | p < 0.01 | p = 0.14 | p = 0.02 | p = 0.01 | p = 0.69 |
| P for trend | p < 0.01 | p < 0.01 | p = 0.06 | p = 0.01 | p = 0.02 | p = 0.56 |
| **Cycling** |  |  |  |  |  |  |
| **Groups** |  |  |  |  |  |  |
| Low | ref | ref | ref | ref | ref | ref |
| Moderate | 0.83[0.65, 1.06] | 0.81[0.63, 1.03] | 0.88[0.68, 1.13] | 0.32[0.15, 0.61] | 0.27[0.12, 0.55] | 0.31[0.14, 0.63] |
|  | p = 0.14 | p = 0.09 | p = 0.31 | p < 0.01 | p < 0.01 | p < 0.01 |
| High | 0.57[0.43, 0.75] | 0.55[0.41, 0.73] | 0.64[0.48, 0.86] | 0.23[0.09, 0.50] | 0.19[0.07, 0.42] | 0.24[0.09, 0.56] |
|  | p < 0.01 | p < 0.01 | p < 0.01 | p < 0.01 | p < 0.01 | p < 0.01 |
| P for trend | p < 0.01 | p < 0.01 | p < 0.01 | p < 0.01 | p < 0.01 | p < 0.01 |
| **Heavy domestic work** |  |  |  |  |  |  |
| **Groups** |  |  |  |  |  |  |
| Low | ref | ref | ref | ref | ref | ref |
| Moderate | 0.74[0.58, 0.94] | 0.74[0.58, 0.95] | 0.71[0.51, 0.99] | 0.57[0.31, 0.99] | 0.51[0.27, 0.91] | 0.54[0.24, 1.25] |
|  | p = 0.02 | p = 0.02 | p = 0.04 | p = 0.06 | p = 0.03 | p = 0.15 |
| High | 0.55[0.41, 0.73] | 0.54[0.40, 0.72] | 0.54[0.37, 0.78] | 0.41[0.18, 0.81] | 0.34[0.14, 0.69] | 0.38[0.14, 1.01] |
|  | p < 0.01 | p < 0.01 | p < 0.01 | p = 0.02 | p < 0.01 | p = 0.06 |
| P for trend | p < 0.01 | p < 0.01 | p < 0.01 | p < 0.01 | p < 0.01 | p = 0.06 |
| **Light domestic work** |  |  |  |  |  |  |
| **Groups** |  |  |  |  |  |  |
| Low | ref | ref | ref | ref | ref | ref |
| Moderate | 0.80[0.64, 1.01] | 0.80[0.63, 1.01] | 1.33[0.80, 2.24] | 0.58[0.34, 0.97] | 0.52[0.29, 0.90] | 0.73[0.25, 2.33] |
|  | p = 0.06 | p = 0.06 | p = 0.27 | p = 0.04 | p = 0.02 | p = 0.59 |
| High | 0.81[0.63, 1.03] | 0.80[0.62, 1.02] | 1.53[0.89, 2.65] | 0.58[0.30, 1.02] | 0.56[0.28, 1.04] | 0.84[0.25, 3.01] |
|  | p = 0.08 | p = 0.08 | p = 0.12 | p = 0.07 | p = 0.08 | p = 0.79 |
| P for trend | p = 0.05 | p = 0.05 | p = 0.02 | p = 0.03 | p = 0.03 | p = 0.78 |
| **Gardening** |  |  |  |  |  |  |
| **Groups** |  |  |  |  |  |  |
| Low | ref | ref | ref | ref | ref | ref |
| Moderate | 0.82[0.60, 1.10] | 0.82[0.59, 1.10] | 0.88[0.64, 1.20] | 0.48[0.18, 1.04] | 0.40[0.15, 0.89] | 0.48[0.17, 1.09] |
|  | p = 0.19 | p = 0.20 | p = 0.42 | p = 0.09 | p = 0.04 | p = 0.11 |
| High | 0.61[0.42, 0.85] | 0.62[0.43, 0.87] | 0.71[0.49, 1.01] | 0.35[0.11, 0.86] | 0.32[0.09, 0.80] | 0.42[0.12, 1.08] |
|  | p < 0.01 | p < 0.01 | p = 0.06 | p = 0.04 | p = 0.03 | p = 0.11 |
| P for trend | p < 0.01 | p = 0.01 | p = 0.06 | p = 0.02 | p = 0.01 | p = 0.04 |
| **Walking** |  |  |  |  |  |  |
| **Groups** |  |  |  |  |  |  |
| Low | ref | ref | ref | ref | ref | ref |
| Moderate | 1.13[0.89, 1.43] | 1.11[0.87, 1.40] | 1.11[0.88, 1.42] | 1.72[1.01, 3.00] | 1.54[0.87, 2.76] | 1.40[0.78, 2.55] |
|  | p = 0.31 | p = 0.39 | p = 0.37 | p = 0.05 | p = 0.14 | p = 0.26 |
| High | 1.01[0.80, 1.28] | 1.00[0.79, 1.26] | 1.01[0.79, 1.29] | 1.40[0.80, 2.48] | 1.20[0.66, 2.19] | 1.06[0.57, 1.97] |
|  | p = 0.92 | p = 0.98 | p = 0.92 | p = 0.24 | p = 0.56 | p = 0.86 |
| P for trend | p = 0.95 | p = 0.97 | p = 0.92 | p = 0.27 | p = 0.58 | p = 0.88 |

Note. PA: Physical activity; CI: Confidence.

^a^Model 0: adjusted for sex, age, and Rotterdam Study Cohort.

^b^Model 1: adjusted for sex, age, Rotterdam Study Cohort, BMI, alcohol intake, smoking and educational level.

^c^Model 2: adjusted for sex, age, Rotterdam Study Cohort, BMI, alcohol intake, smoking, educational level and the other subtype physical activities.

^d^ Probable sarcopenia and sarcopenia were categorized according to EWGSOP2 criteria (Table 1). Probable sarcopenia was also defined as low grip strength according to EWGSOP2 cut-off points.

^e^The odds ratios represent the risk for probable sarcopenia and sarcopenia compared to non-sarcopenic individuals across different physical activity types.

| **Supplementary table 8. Sex-stratified analysis between total physical activity and specific physical activity types with continuous ALMI and GS in males.** | | | | | | |
| --- | --- | --- | --- | --- | --- | --- |
|  | **Appendicular lean mass index**  **(Model 0^a^)** | **Appendicular lean mass index**  **(Model 1^b^)** | **Appendicular lean mass index**  **(Model 2^c^)** | **Grip strength**  **(Model 0^a^)** | **Grip strength**  **(Model 1^b^)** | **Grip strength**  **(Model 2^c^)** |
| **β ^d^ [CI 95%]** | | | | | | |
| **Total PA** | 0.018[0.011, 0.025] | 0.021[0.016, 0.026] |  | 0.139[0.078, 0.199] | 0.142[0.081, 0.203] |  |
|  | p < 0.001 | p < 0.001 |  | p < 0.001 | p < 0.001 |  |
| **Sports** | 0.025[0.013, 0.036] | 0.026[0.018, 0.035] | 0.018[0.009, 0.027] | 0.207[0.105, 0.309] | 0.215[0.112, 0.317] | 0.179[0.070, 0.288] |
|  | p < 0.001 | p < 0.001 | p < 0.001 | p < 0.001 | p < 0.001 | p = 0.001 |
| **Cycling** | 0.060[0.024, 0.096] | 0.076[0.050, 0.103] | 0.057[0.030, 0.084] | 0.370[0.054, 0.686] | 0.395[0.078, 0.713] | 0.240[-0.087, 0.568] |
|  | p = 0.001 | p < 0.001 | p < 0.001 | p = 0.022 | p = 0.015 | p = 0.150 |
| **Heavy domestic work** | 0.051[0.025, 0.077] | 0.051[0.032, 0.070] | 0.039[0.019, 0.059] | 0.464[0.237, 0.691] | 0.489[0.261, 0.717] | 0.465[0.223, 0.706] |
|  | p < 0.001 | p < 0.001 | p < 0.001 | p < 0.001 | p < 0.001 | p < 0.001 |
| **Light domestic work** | 0.039[0.012, 0.065] | 0.034[0.015, 0.053] | 0.007[-0.014, 0.027] | 0.095[-0.136, 0.326] | 0.103[-0.130, 0.336] | -0.161[-0.411, 0.088] |
|  | p = 0.004 | p < 0.001 | p = 0.514 | p = 0.422 | p = 0.387 | p = 0.205 |
| **Gardening** | 0.031[0.004, 0.059] | 0.032[0.012, 0.052] | 0.028[0.008, 0.047] | 0.067[-0.174, 0.307] | 0.074[-0.167, 0.315] | 0.043[-0.197, 0.283] |
|  | p = 0.026 | p = 0.002 | p = 0.006 | p = 0.586 | p = 0.546 | p = 0.727 |
| **Walking** | -0.012[-0.036, 0.012] | 0.005[-0.013, 0.023] | -0.004[-0.022, 0.014] | 0.097[-0.114, 0.309] | 0.057[-0.162, 0.276] | -0.009[-0.229, 0.211] |
|  | p = 0.329 | p = 0.585 | p = 0.658 | p = 0.366 | p = 0.609 | p = 0.936 |

Note. PA: Physical activity; CI: Confidence interval.

^a^Model 0: adjusted for age, and Rotterdam Study Cohort.

^b^Model1: adjusted for age, Rotterdam Study Cohort, BMI, alcohol intake, smoking and educational level.

^c^Model2: adjusted for age, Rotterdam Study Cohort, BMI, alcohol intake, smoking, educational level and the other subtype physical activities.

^d^The β and odds ratios are per 10 MET*hours*week^-1^ increase in any of the physical activity types for the continuous analysis.

| **Supplementary table 9. Sex-stratified analysis between total physical activity and specific physical activity types with continuous ALMI and GS in females.** | | | | | | |
| --- | --- | --- | --- | --- | --- | --- |
|  | **Appendicular lean mass index**  **(Model 0^a^)** | **Appendicular lean mass index**  **(Model 1^b^)** | **Appendicular lean mass index**  **(Model 2^c^)** | **Grip strength**  **(Model 0^a^)** | **Grip strength**  **(Model 1^b^)** | **Grip strength**  **(Model 2^c^)** |
|  | **β ^d^ [CI 95%]** | **β [CI 95%]** | **β [CI 95%]** | **β [CI 95%]** | **β [CI 95%]** | **β [CI 95%]** |
| **Total PA** | -0.001[-0.006, 0.004] | 0.011[0.007, 0.014] |  | 0.077[0.046, 0.108] | 0.085[0.054, 0.117] |  |
|  | p = 0.755 | p < 0.001 |  | p < 0.001 | p < 0.001 |  |
| **Sports** | 0.000[-0.011, 0.010] | 0.018[0.011, 0.025] | 0.013[0.006, 0.020] | 0.140[0.073, 0.206] | 0.143[0.076, 0.211] | 0.109[0.039, 0.180] |
|  | p = 0.934 | p < 0.001 | p < 0.001 | p < 0.001 | p < 0.001 | p = 0.002 |
| **Cycling** | 0.039[0.009, 0.070] | 0.073[0.053, 0.093] | 0.063[0.043, 0.084] | 0.375[0.181, 0.570] | 0.391[0.196, 0.586] | 0.306[0.108, 0.504] |
|  | p = 0.010 | p < 0.001 | p < 0.001 | p < 0.001 | p < 0.001 | p = 0.002 |
| **Heavy domestic work** | 0.006[-0.012, 0.024] | 0.023[0.011, 0.035] | 0.016[0.002, 0.030] | 0.155[0.038, 0.272] | 0.182[0.065, 0.299] | 0.123[-0.011, 0.256] |
|  | p = 0.520 | p < 0.001 | p = 0.021 | p = 0.009 | p = 0.002 | p = 0.072 |
| **Light domestic work** | -0.002[-0.014, 0.010] | 0.012[0.004, 0.020] | -0.001[-0.010, 0.008] | 0.074[-0.003, 0.151] | 0.091[0.013, 0.168] | -0.005[-0.096, 0.085] |
|  | p = 0.725 | p = 0.004 | p = 0.811 | p = 0.059 | p = 0.023 | p = 0.907 |
| **Gardening** | -0.002[-0.030, 0.025] | 0.027[0.008, 0.045] | 0.020[0.001, 0.038] | 0.199[0.024, 0.375] | 0.193[0.015, 0.372] | 0.152[-0.027, 0.331] |
|  | p = 0.858 | p = 0.004 | p = 0.035 | p = 0.026 | p = 0.034 | p = 0.097 |
| **Walking** | -0.018[-0.034, -0.002] | 0.002[-0.009, 0.013] | -0.002[-0.013, 0.009] | 0.077[-0.027, 0.181] | 0.096[-0.008, 0.200] | 0.067[-0.037, 0.171] |
|  | p = 0.024 | p = 0.704 | p = 0.726 | p = 0.146 | p = 0.070 | p = 0.204 |

Note. PA: Physical activity; CI: Confidence interval.

^a^Model 0: adjusted for age, and Rotterdam Study Cohort.

^b^Model1: adjusted for age, Rotterdam Study Cohort, BMI, alcohol intake, smoking and educational level.

^c^Model2: adjusted for age, Rotterdam Study Cohort, BMI, alcohol intake, smoking, educational level and the other subtype physical activities.

^d^The β and odds ratios are per 10 MET*hours*week^-1^ increase in any of the physical activity types for the continuous analysis.

| **Supplementary table 10. Multicollinearity of variables** | |
| --- | --- |
| **Variables** | **GVIF*** |
| **Sports** | 1.15 |
| **Cycling** | 1.07 |
| **Heavy domestic work** | 1.26 |
| **Light domestic work** | 1.38 |
| **Gardening** | 1.02 |
| **Walking** | 1.03 |
| **Sex** | 1.27 |
| **Age** | 2.96 |
| **Rotterdam Study Cohort** | 2.86 |
| **BMI** | 1.04 |
| **Alcohol intake** | 1.13 |
| **Smoking** | 1.13 |
| **Educational level** | 1.18 |

*GVIF (Generalized Variance Inflation Factor): A measure of multicollinearity for each predictor. Higher GVIF values indicate more severe multicollinearity. A GVIF value above 5 suggests potential multicollinearity, where the predictor may be strongly correlated with others in the model.

**Supplementary Figure 1.** **Spearman correlation matrix between different physical activity types and sarcopenia components.**


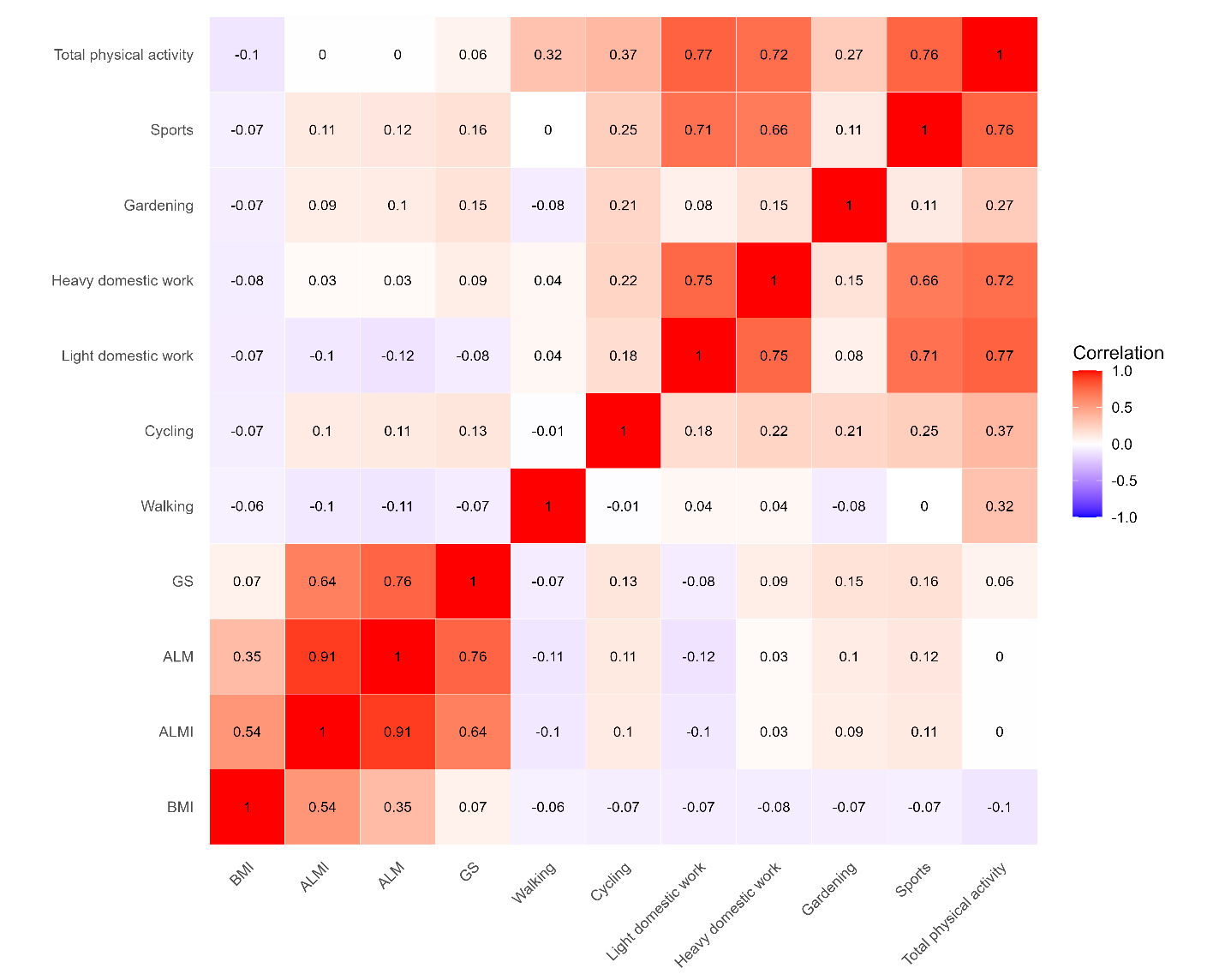


**Supplementary Figure 2. Flow chart.**

Participants without DXA data

N = 1198

Participants without physical activity data N = 751

Participants without hand grip data

N = 76

Participants without weight, height and informed consent information

N = 27

Participants N = 4849

Participants N = 5133

Participants N = 5884

Participants N = 5960

All participants in the fifth wave of the Rotterdam Study

N = 7162

Participants N = 5106

Participants without covariates data (alcohol intake, smoking and educational level, n = 84)

Complete data N = 4765

Participants with BMI > 35

N = 257

**Supplementary Figure 3a. Number and proportion of individuals with probable sarcopenia and sarcopenia allocated in physical activity groups**.
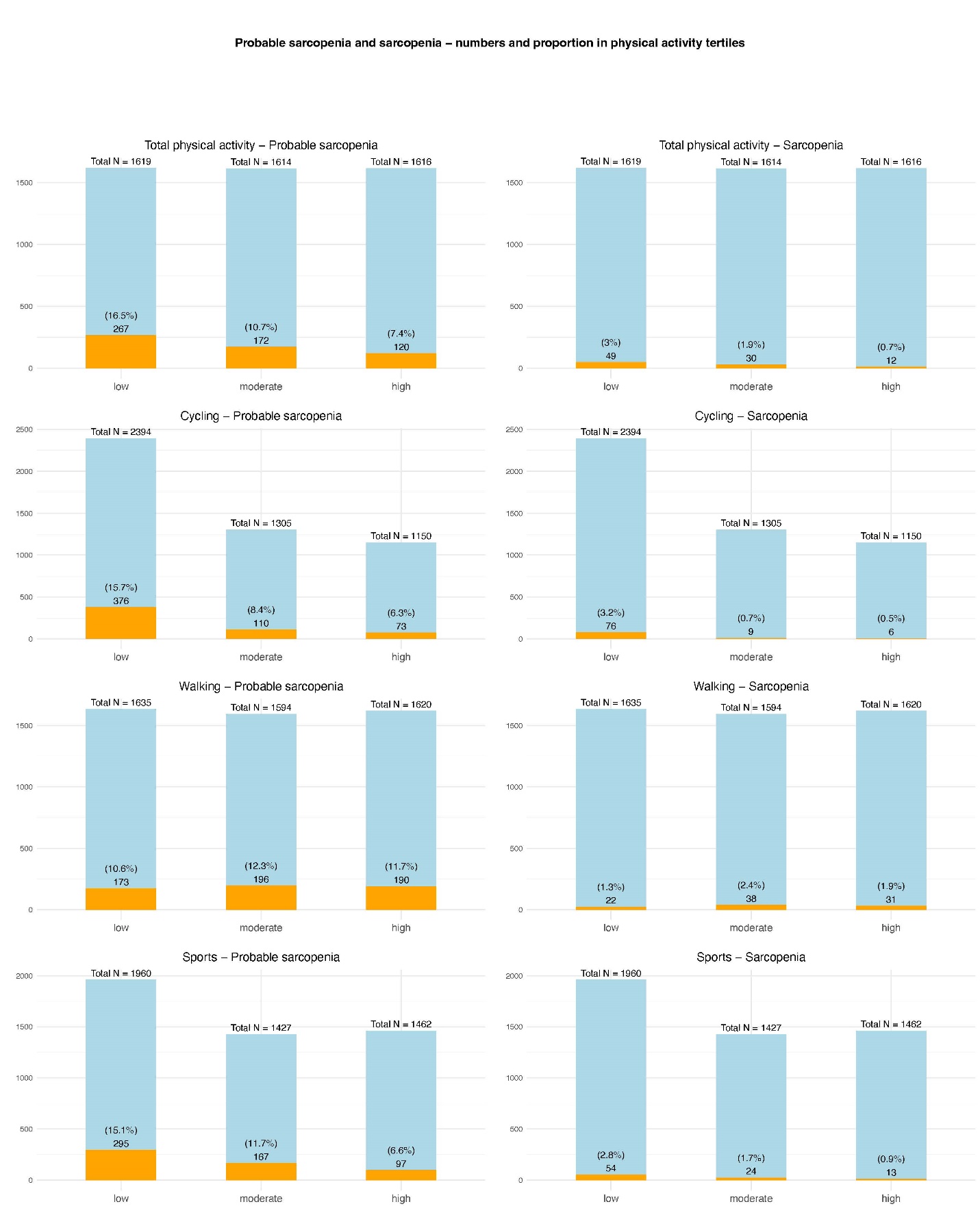


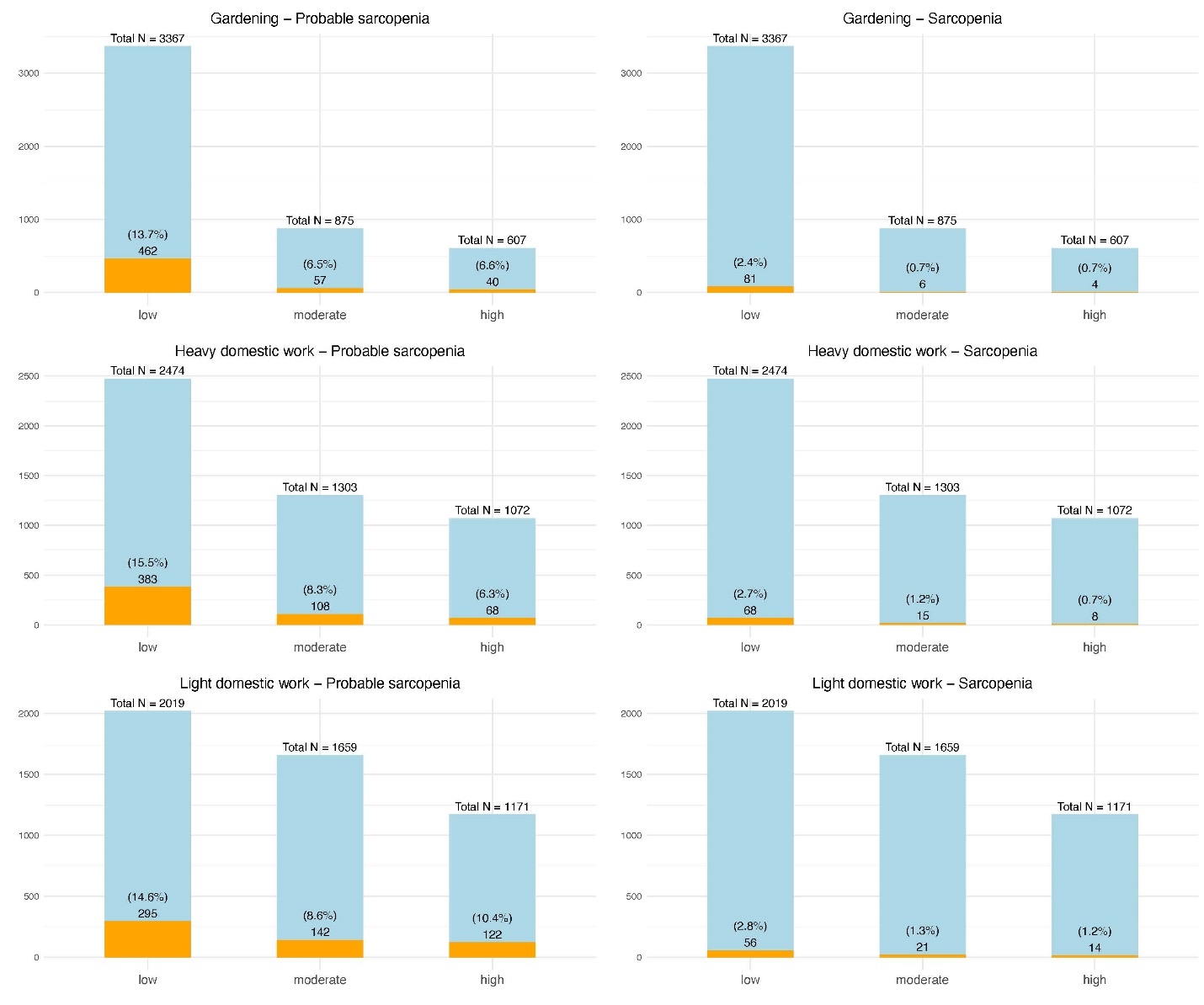


**Supplementary Figure 3b. Number and proportion of individuals with low ALMI allocated in physical activity groups.**
